# Comprehensive assessment of measurement uncertainty in ^13^C-based metabolic flux experiments

**DOI:** 10.1007/s00216-018-1017-7

**Published:** 2018-04-13

**Authors:** Teresa Mairinger, Wolfhard Wegscheider, David Alejandro Peña, Matthias G. Steiger, Gunda Koellensperger, Jürgen Zanghellini, Stephan Hann

**Affiliations:** 1 Department of Chemistry, University of Natural Resources and Life Sciences, Muthgasse 18, 1190 Vienna, Austria; 2 Department of General, Analytical and Physical Chemistry, University of Leoben, Franz-Josef-Strasse 18, 8700 Leoben, Austria; 3 Austrian Centre of Industrial Biotechnology, Muthgasse 11, 1190 Vienna, Austria; 4 Department of Biotechnology, University of Natural Resources and Life Sciences, Muthgasse 18, 1190 Vienna, Austria; 5 Institute of Analytical Chemistry, University of Vienna, Währinger Strasse 38, 1090 Vienna, Austria

**Keywords:** Measurement uncertainty, Metabolic flux analysis, Metabolic engineering, Isotopologue analysis, Isotope interference correction

## Abstract

In the field of metabolic engineering ^13^C-based metabolic flux analysis experiments have proven successful in indicating points of action. As every step of this approach is affected by an inherent error, the aim of the present work is the comprehensive evaluation of factors contributing to the uncertainty of nonnaturally distributed C-isotopologue abundances as well as to the absolute flux value calculation. For this purpose, a previously published data set, analyzed in the course of a ^13^C labeling experiment studying glycolysis and the pentose phosphate pathway in a yeast cell factory, was used. Here, for isotopologue pattern analysis of these highly polar metabolites that occur in multiple isomeric forms, a gas chromatographic separation approach with preceding derivatization was used. This rendered a natural isotope interference correction step essential. Uncertainty estimation of the resulting C-isotopologue distribution was performed according to the EURACHEM guidelines with Monte Carlo simulation. It revealed a significant increase for low-abundance isotopologue fractions after application of the necessary correction step. For absolute flux value estimation, isotopologue fractions of various sugar phosphates, together with the assessed uncertainties, were used in a metabolic model describing the upper part of the central carbon metabolism. The findings pinpointed the influence of small isotopologue fractions as sources of error and highlight the need for improved model curation.

**Electronic supplementary material:**

The online version of this article (10.1007/s00216-018-1017-7) contains supplementary material, which is available to authorized users.

**Graphical abstract:**
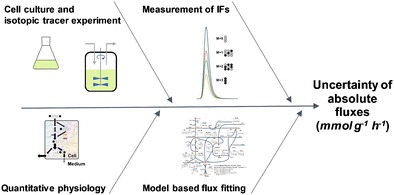
ᅟ

## Introduction

The assessment of absolute intracellular fluxes (i.e., reaction rates per unit cell volume or mass) found its way into metabolic engineering several decades ago. In metabolic engineering, possibilities to influence metabolic reaction rates are of special interest, since the quantitative understanding of metabolic flux regulation mechanisms allows a more precise reengineering of cell factories [1–3]. This technique can be used to engineer organisms such as bacteria or fungi to improve the industrial production of, for example, organic acids [4], lipids [5], or proteins [6]. The estimation of metabolic fluxes relies on stable isotope labeling experiments, where an isotope tracer (e.g., specifically ^13^C labeled glucose) is fed to an organism of interest. The resulting incorporation of the stable label into downstream metabolites is most commonly measured by mass spectrometry (MS)-based methods [3, 7]. Before the detection of these nonnaturally distributed ^13^C labeling patterns of *free* intracellular metabolites, separation of the analytes of interest is indispensable. For this purpose, either liquid chromatographic or gas chromatographic techniques are applied. Although the latter require a laborious derivatization step before analysis, the application of gas chromatography (GC)-based methods is convincing because of the excellent separation efficiency and broad metabolite coverage. This includes the separation of amino acids, organic acids, and metabolites with multiple structural isomeric forms, such as sugars and sugar phosphates, in one analytical run [8–11]. Sugar phosphates are of particular interest since many metabolites of the central carbon metabolism are phosphorylated, and the reaction involved therein often represents points of action in metabolic engineering [12].

As intermediate results of the ^13^C labeling pattern analysis, isotopologue fractions (IFs)—that is, molecular entities that differ in their isotopic composition [13]—of metabolites are obtained [3, 7]. However, the measured distributions do not reflect the true ^13^C labeling patterns, which are the result of metabolizing the tracer molecule, and are interfered with naturally abundant heavy stable isotopes either present in the native molecule itself (e.g., ^34^S) or introduced by derivatization (e.g., alkoxymation and silylation) [7, 14, 15]. Here, the latter is of major influence and concerns mainly interferences of naturally abundant ^13^C, ^29^Si, and ^30^Si. Heavy stable isotopes of elements such as hydrogen, nitrogen, and oxygen have a rather marginal impact because of their low natural abundances. These isotope interferences need to be corrected for to obtain an unbiased result for ^13^C labeling patterns. Several software packages are available for this purpose [14–20].

Together with data on growth, uptake, and secretion rates, as well as the biomass composition of the organism, the isotope-interference-corrected C-isotopologue distributions of metabolites are implemented in biochemical network models. By maximizing the fit between the a priori simulated labeling patterns and the experimentally obtained IFs, one derives intracellular flux values. Finally, statistical analysis of the goodness of fit is performed, and nonlinear confidence intervals for fluxes are computed [21].

The successful outcome in terms of precise and accurate fluxes depends greatly on the quality of the mathematical model describing the metabolic network, on the design of the isotope labeling experiment (namely, the selection of suitable tracers), and on the analysis of labeling patterns [21, 22]. Hence, a careful validation of every single step of the measurement procedure, including the estimation of measurement uncertainty, is highly valuable to generate data of the required quality. Generally, measurement uncertainty is defined as "a non-negative parameter characterizing the dispersion of the quantity values being attributed to a measurand based on the information used” [23]. Thus, measurement uncertainty is a quantitative descriptor of the reliability of a measurement, and states an interval that includes the values that the measurand could reasonably take with a specified probability [23, 24]. With the help of this theoretical concept of measurement uncertainty budgeting, including the quantitative assessment of the contribution of different uncertainty components, method limitations as well as points of action for improvement can be identified [24].

To demonstrate the use of this assessment, a previously published ^13^C-based metabolic flux analysis (MFA) experiment involving a yeast cell factory, namely, *Pichia pastoris* [12, 25], was used as a test data set. The objective of this MFA experiment was an increase in the titer of a model protein (human superoxide dismutase, hSOD). For that purpose, the pentose phosphate pathway (PPP), which is responsible for the formation of reduced NADPH, an important cofactor for the production of metabolites and proteins [12], and which branches off at glucose 6-phosphate (G6P) and runs in parallel to glycolysis, was engineered by overexpression of different PPP genes. By use of MFA, it was demonstrated, that the combined overexpression of glucose 6-phosphate dehydrogenase gene (*ZWF1*) and 6-phosphogluconolactonase gene (*SOL3*) enhanced the flux through the PPP and led to an increased yield of hSOD [12]

The evaluation of the reliability of analytical results is of core importance as key decisions are taken on their basis. In the present study, measurement uncertainty budgeting according to EURACHEM’s *Quantifying Uncertainty in Analytical Measurement* guidelines [24] was performed to evaluate major factors contributing to the uncertainty of isotopologue analysis. For that purpose, after identification of the measurand and its contributing influencing factors, a model equation that considers all uncertainty components relevant to the result was set up, and Monte Carlo simulation was applied for error modeling and propagation. More precisely, the distribution of the measurement results was obtained by randomly changing the input parameters within their standard uncertainties and probability function. As an outcome, the contribution of all probability density functions of the uncertainty components involved was visualized [26].

The isotopologue distributions of the different metabolites, including the modeled uncertainties, were then used in the metabolic model to investigate the robustness of the model as well as the impact of uncertainty and precision on the calculated flux values. We highlight that despite an elaborate body of theory on error propagation in MFA [27], the impact of the underlying metabolic models and the low-abundance IFs as a source of error has been underestimated.

To the best of our knowledge, this is the first time Monte Carlo simulation has been used for a comprehensive assessment of the measurement uncertainty of IFs, and its influence on the outcome of absolute flux values was investigated.

## Materials and methods

### Isotopologue distribution analysis

A ^13^C-based MFA experiment targeting the branching point of glycolysis and the PPP in *P. pastoris* [12, 25] was used as a model data set for the assessment of measurement uncertainty. In this MFA experiment, possibilities to increase the titer of a recombinant protein (hSOD) in a yeast cell factory were studied with PPP gene overexpression. As isotopic tracer [1,6-^13^C_2_]glucose was used, and the resulting labeling patterns of intracellular metabolites were measured by a gas chromatographic approach with soft ionization (namely, chemical ionization) coupled with high-resolution time-of-flight (TOF) MS. Full details on the cultivation conditions, the analytical method, the essential natural isotope interference correction, and the isotopologue distributions used in this study can be found in [25]. For absolute flux value calculation, isotopologue information on glucose-6-phosphate (G6P), glyceraldehyde 3-phosphate (GAP), dihydroxyacetone phosphate (DHAP), erythrose 4-phosphate (E4P), ribulose 5-phosphate (Rl5P), ribose 5-phosphate (R5P), fructose 6-phosphate (F6P), and sedoheptulose 7-phosphate (S7P) was used as input data.

### Measurement uncertainty assessment using Monte Carlo simulation

The Microsoft Excel add-in @RISK (version 7.5.1; Palisade, Ithaca, NY, USA) was used for Monte Carlo simulation. The definition of uncertainty components and their respective distributions can be found in Table 1. One hundred thousand iterations were run for one simulation. Since interference correction of naturally occurring heavy stable isotopes is a prerequisite here, the correction step for ^13^C, ^29^Si, and ^30^Si was included in measurement uncertainty budgeting and applied to the measured IFs of *P. pastoris*, published in [25]. Expressions for isotope interference correction of the respective metabolites are listed in Table S1.

**Table 1:** Description of uncertainty components, including unit, standard uncertainty, and their respective distribution

Uncertainty component	Quantity	Definition	Standard uncertainty	Distribution
${A}_{n_{\mathrm{raw}}}$	Ion counts	Integrated *raw* area of the M+*n* isotopologue of the investigated molecule containing *n* ^13^C atoms	$\sqrt{\mathrm{Ion}\ \mathrm{counts}}$	Poisson
*f*_int_	Factor	Estimated reliability of automated peak integration corrected for ion counting statistics	2.0%	Triangular
*f*_ion_	Factor	Precision of ionization and ion transmission process	Specific	Normal
*A*_*n*_	Intermediate result	Area to be used for isotope interference correction, including uncertainty contributions of ion counting statistics, precision of peak integration, and precision of ionization and ion transmission	Specific	Normal
${A}_{n_{\mathrm{corr}}}$	Isotope-interference-corrected areas	Areas corrected for isotope interferences of naturally distributed carbon and silicon; in case of the exemplary compound Rl5P 5 trimethylsilyl groups and 1 ethoxime group (in total 16 carbon atoms and 5 silicon a toms)	Specific	Normal
*F*_*n*_	Logic variable	Binary variable being applied because of overcorrection. For negative values after interference correction, the resulting area is set to zero by multiplication by 0. Positive values are not affected as they are multiplied by 1		
*a*	Normalized natural isotope abundance of ^30^Si	Isotope abundance of ^30^Si, normalized to the abundance of ^28^Si → 0.03353 (*μ* = 0.03092)	0.000055	Normal
*b*	Normalized natural isotope abundance of ^29^Si	Isotope abundance of ^29^Si, normalized to the abundance of ^28^Si → 0.05080 (*μ* = 0.04685)	0.00004	Normal
*c*	Normalized natural isotope abundance of ^13^C	Isotope abundance of ^13^C, normalized to the abundance of ^12^C → 0.01082 (*μ* = 0.0107)	0.0004	Normal
${\mathrm{IF}}_{\mathrm{M}+{1}_{\mathrm{corr}}}$	Ratio	Calculated IF of M+1 of a molecule containing 5 carbons in the backbone calculated from isotope-interference-corrected peak areas A_0corr_ to A_5corr_		

### Flux and confidence interval estimation

Metabolic fluxes were estimated with the toolbox OpenFlux version 2.1 implemented in MATLAB 2015b [28]. The metabolic network of glycolysis and the PPP of *P. pastoris* used was modified from a previously published version [25]. The lower part of glycolysis was removed to simplify the model and focus on the ratio of interest: glycolysis/PPP. An OpenFlux-compatible version of the model is available in the electronic supplementary material. Confidence intervals were estimated by Monte Carlo simulations with 10,000 samples obtained by our corrupting the measurements with (normally distributed) noise within the provided uncertainty of measurement. Statistical analysis was also performed in MATLAB as described in [27] with use of a modified version of the toolbox OpenFlux2 version 1.2.4 [29]. The goodness of fit was tested by our comparing the minimized variance-weighted sum of squared residuals with a X^2^-distributed stochastic variable, using a 95% confidence level and a degree of freedom equal to the number of independent measurements minus the number of free fluxes. The metabolic network was drawn with Escher [30].

## Results and discussion

**Fig. 1:**
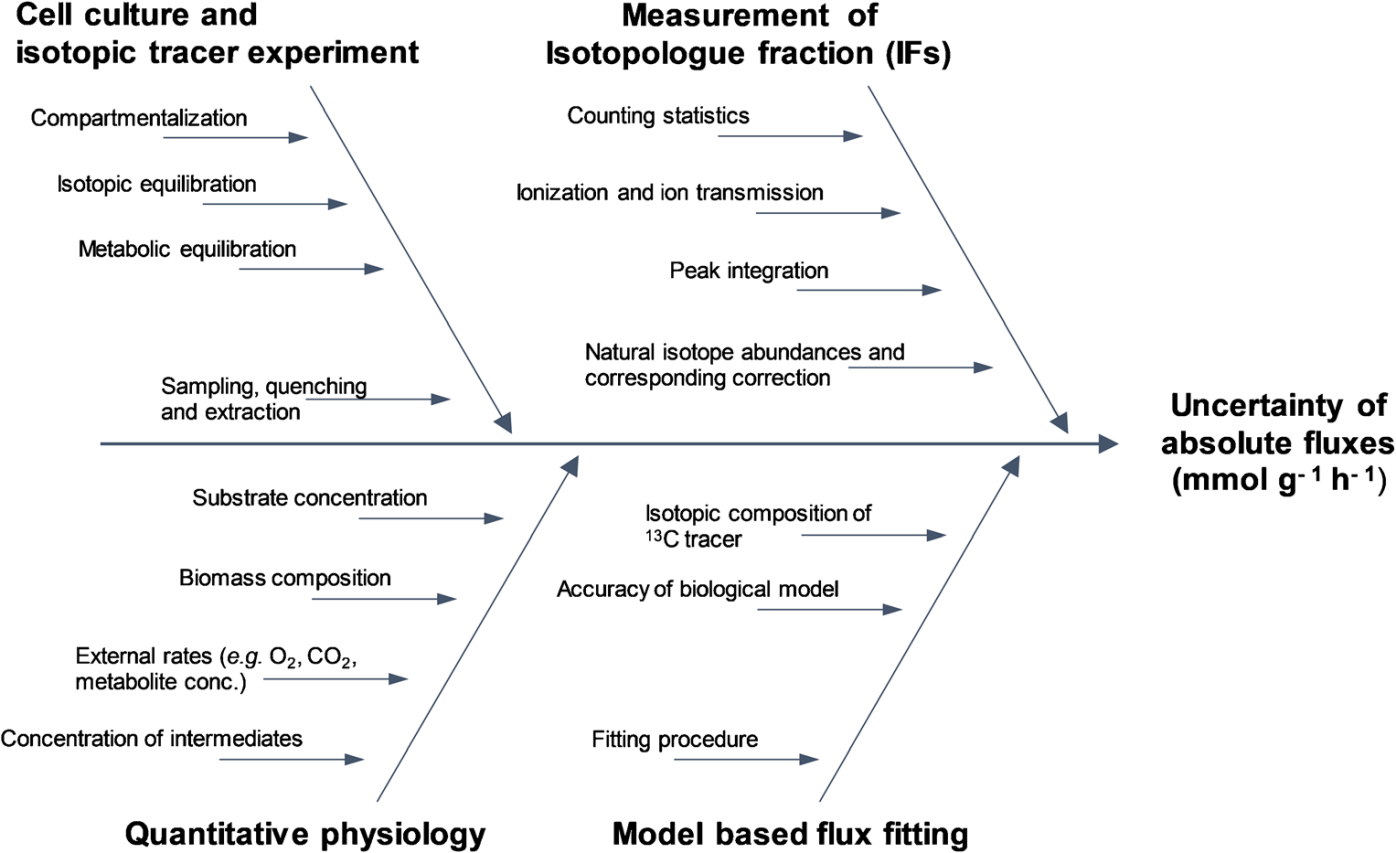
Ishikawa diagram (also known as a “cause-and-effect diagram”) for identification of possible sources of measurement uncertainty of absolute flux values showing the most critical contributors to the uncertainty of absolute fluxes

After the measurand has been specified, in the next step of the uncertainty estimation process different uncertainty sources contributing to the total uncertainty of absolute flux values need to be identified, and can be visualized in a cause-and-effect diagram, as shown in Fig. 1 [24].

### Identification of influencing factors contributing to measurement uncertainty of metabolic fluxes

#### Contribution of the biological variability

The general biological variability is commonly estimated as approximately 15% [31], and hence has a major influence on uncertainty. Since the cell is highly complex in terms of its metabolism, controlling the metabolic state during the experiment is vital. Importantly, ^13^C flux experiments are generally not performed on a single-cell level. However, to obtain reliable results, a cell population needs to behave homogeneously regarding its metabolic flux distribution, but also in terms of its genetic stability. In general, it is assumed that a metabolic steady state can be obtained when the growth rate is constant, which holds true in a chemostat and with some care taken also during the exponential growth of batch or fed-batch cultures [1]. Other factors worth mentioning are the medium composition and other environmental impacts during cultivation, such as light, gassing, or inoculum size. The biological sources of uncertainty can be roughly differentiated into influencing factors stemming from the actual isotope labeling experiment on the respective cell culture and aspects of quantitative cell physiology. A rather general point to be considered in the case of eukaryotes is the presence of one metabolite in different cell compartments. However, the last point can be ignored in the present study, since the metabolic reactions investigated all occur in the cytosol. Besides, sampling and quenching protocols might introduce further variability [32]. Since reactions in the *upper* part of the metabolic network (i.e., glycolysis and PPP) are characterized by high turnover rates, stopping of all metabolic activities before sampling needs to be scrutinized [33]. Apart from compound-specific physicochemical stability, this interplay between quenching/sampling procedures and differences in metabolic turnover rates leads to metabolite-specific biological variations. To indicate this exemplarily, isotope-interference-corrected IFs of alanine and G6P are compared in terms of their biology- and measurement-based uncertainty in Fig. S1. In general, the different biological variances are hard to assess in an isolated manner since they are not independent observables. Hence, for practical reasons, the precision obtained under repeatability conditions of measurement of three biological replicates of the described tracer experiment [25] was used as the standard uncertainty for the IFs of the different metabolites.

#### Contribution of the analytical measurement

Another contribution to the uncertainty of absolute fluxes is the actual analytical measurement procedure involved in obtaining the ^13^C labeling patterns. As stated before, different analytical approaches can be used for the measurement. In this study, a GC–MS approach was used since the chromatographic separation of the multiple isomers of the different sugar phosphates is crucial. Influencing factors relevant to this measurement procedure are depicted in the fishbone diagram in Fig. 1. Clearly, ion counting statistics are relevant, especially in the case of low-abundance isotopologues with low signal intensity. Besides ionization, ion transmission of isotopologues within the mass spectrometer needs to be accounted for. The assessment of isotopologue distribution is most commonly based on integration of the chromatographic peak areas of the respective isotopologues. In general, peak integration is performed automatically according to dedicated algorithms. However, because of noise or potential interferences, both being possibly present in a complex biological matrix, the peak integration process, including appropriate baseline recognition, is prone to errors, and needs to be checked also manually. Additionally, in ^13^C-based MFA, the integration of each of the isotopologues of a metabolite needs to be highly repeatable concerning the respective integration limits. This especially holds true for low-abundance signals or signals affected by a high background level as they are more affected by small imprecisions on integrated peak areas. Besides, because of the necessary derivatization procedure in GC, another uncertainty component needs to be defined; that is*,* the variation of the natural isotope composition and the corresponding error propagation due to the isotope interference correction process.

#### Contribution of estimating metabolic flux values

The uncertainty in fluxes is also influenced by the assumed structure of the underlying metabolic network. During the experimental design, fluxes of interest are selected, and a labeled substrate is identified so that the desired fluxes can be resolved [21]. This is performed by simulation of the resulting labeling patterns with the metabolic model [27], [34]. Thus, it is critical to include all reactions that may significantly affect the isotopologue distribution of the measured metabolites and verify their atom transitions and (ir)reversibilities. Errors during the network reconstruction may result in inappropriate fitting of the measurement data, which could translate into inaccurate or imprecise fluxes. Specifically, the computed flux distribution results from the minimization of uncertainty-weighted residuals between the measured and the simulated IFs. However, if the model becomes too complex (e.g., by allowing too many reversible steps or reactions), the statistical significance of the estimated fluxes decreases, unless more data are provided.

## Assessment of measurement uncertainty of isotopologue analysis

As an example, for the measurement uncertainty of the C-isotopologue distribution, the model equation for the isotope-interference-corrected IF M+1 $\left({\mathrm{IF}}_{\mathrm{M}+{1}_{\mathrm{corr}}}\right)$ of a metabolite, namely, Rl5P, is described in Eq. 1.

1 $$ {\mathrm{IF}}_{M+{1}_{\mathrm{corr}}}=\frac{A_{1_{\mathrm{corr}}}\times {F}_1}{A_0+{A}_{1_{\mathrm{corr}}}\times {F}_1+{A}_{2_{\mathrm{corr}}}\times {F}_2+{A}_{3_{\mathrm{corr}}}\times {F}_3+{A}_{4_{\mathrm{corr}}}\times {F}_4+{A}_{5_{\mathrm{corr}}}\times {F}_5} $$

This specific IF of Rl5P contains five carbon atoms in the backbone, of which four carbons are ^12^C isotopes and one is a ^13^C isotope. The uncertainty components, including their standard uncertainties and assigned distributions, are described in Table 1.

It cannot be ruled out that certain effects, such as ionization, ion transmission, and peak integration, have different contributions to the measurement uncertainty of the respective IFs. This means that the size of the contribution may vary with peak intensity. To account for this, the integrated peak areas were multiplied by different factors. To keep the integrated peak area unaltered, for these factors a numerical value of 1 was applied and defined by a standard deviation and distribution, as shown in Table 1. To estimate the influence of the contribution of ionization and ion transmission, integrated isotopologue peak areas of various derivatized metabolites were plotted against their respective standard deviations, and the slope of the resulting linear equation was used as the standard uncertainty for the normally distributed *f*_ion_. The peak areas used to set up this linear regression were obtained in the course of method validation, published in [25]. For assessment of the reliability of automated peak integration, a standard uncertainty of 2% of the respective area was estimated from empirical data. This factor (i.e., *f*_int_), however, needs to be additionally corrected for the contribution of ion counting statistics, since this is already accounted for by application of a Poisson distribution to the integrated raw peak areas of the derivative’s isotopologues. Indeed, the uncertainty of automated peak integration can be decreased when manual integration is used; however, since this would be accompanied by a highly operator dependent value, this was not done in order to allow a *standardized* estimation of uncertainty. The equation for this intermediate result is as follows:

2 $$ {A}_n={A}_{n_{\mathrm{raw}}}\times {f}_{\mathrm{int}}\times {f}_{n\_\mathrm{ion}} $$

As mentioned in “Introduction,” the measured IFs are significantly biased by the naturally abundant heavy stable isotopes introduced via the derivatization. For the exemplary compound Rl5P, five trimethylsilyl groups and one ethoxime group are added in this step. Since for sugar phosphates the [M − CH_3_]^+^ ion is used for isotopologue analysis by GC–chemical ionization quadrupole TOF MS [11], this leads to a total of 16 naturally distributed carbon atoms and five silicon atoms, which both impact the ^13^C labeling pattern of the native molecule. Obviously, there are also heavy stable isotopes of hydrogen, nitrogen, and oxygen present in the molecule that contribute to the isotope envelope; however, since their natural abundance is comparatively low (0.0115% D, 0.364% ^15^N, and 0.205% ^18^O), these are omitted from the isotope interference correction. The area of the monoisotopic isotopologue *A*_0_ is not affected by isotopic interferences, whereas the interferences of the other isotopologues increase with increasing mass. Natural isotope interference correction was based on combinatorics and on the known natural abundance of the major interfering elements; namely, carbon and silicon [35]. The set of expressions necessary to correct the isotope interferences of the exemplary compound (i.e., a C_5_ backbone and C_16_Si_5_ to be corrected) is given in Table 2 and was implemented in a Microsoft Excel spreadsheet. Here, *a* represents the normalized isotope abundance of ^30^Si, *b* represents the normalized isotope abundance of ^29^Si, and *c* represents the normalized isotope abundance of ^13^C. Standard uncertainties of these heavy isotopes were taken from an IUPAC technical report [35], and were normalized to the lightest isotope of the respective element. Because of the measurement error, inherent to any analytical procedure, as well as the variance of natural isotope distributions, negative intensities can occur in the correction approach described, as discussed in [14, 17, 19]. In practice, isotope interference correction is hardly performed in Microsoft Excel but is rather done with dedicated software packages. With use of these algorithms, negative values are typically set to zero during the solution process [14, 17, 19]. To account for this in the present approach, a logic variable, *F*_*n*_, was introduced during the isotope interference correction process. This variable sets negative peak area values to zero, whereas positive ones are left unaltered. By use of this pseudofactor, it is also possible to elucidate the uncertainty of these negative values. The described measurement uncertainty budgeting using the Microsoft Excel add-in @Risk of Rl5P is given as electronic supplementary material.

**Table 2:** Expressions for isotope interference correction of ribulose 5-phosphate (Rl5P), a metabolite with 5 carbons in the backbone and 16 carbon atoms and 5 silicon atoms, both being naturally distributed, to be corrected for.

Isotope-inference-corrected area of M+ *n* isotopologue	Expression for correction
*A*_0_	*A*_0_
${A}_{1_{\mathrm{corr}}}$	*A*_1_-(5*b*×*A*_0_)-(16*c*×*A*_0_)
${A}_{2_{\mathrm{corr}}}$	*A*_2_-(5*a*×*A*_0_)-(10*b*^2^×*A*_0_)-(5*b*×${A}_{1_{\mathrm{corr}}}$)-[(16*c*×5*b*)×*A*_0_]-(16*c*$\times {A}_{1_{\mathrm{corr}}}$)-(120*c*^2^×*A*_0_)
${A}_{3_{\mathrm{corr}}}$	*A*_3_-(20*ab*×*A*_0_)-(5*a*$\times {A}_{1_{\mathrm{corr}}}$)-(10*b*^3^×*A*_0_)-(10*b*^2^$\times {A}_{1_{\mathrm{corr}}}$)-(5*b*$\times {A}_{2_{\mathrm{corr}}}$)-[(16*c*×5*a*)×*A*_0_]-[(16*c*×10*b*^2^)×*A*_0_]-[(16*c*×5*b*)×${A}_{1_{\mathrm{corr}}}$]-(16*c*×${A}_{2_{\mathrm{corr}}}$)-[(120*c*^2^×5*b*)×*A*_0_]-(120*c*^2^×${A}_{1_{\mathrm{corr}}}$)-(560*c*^3^×*A*_0_)
${A}_{4_{\mathrm{corr}}}$	*A*_4_-(20*ab*×${A}_{1_{\mathrm{corr}}}$)-(30*ab*^2^×*A*_0_)-(10*a*^2^×*A*_0_)-(5*a*×${A}_{2_{\mathrm{corr}}}$)-(5*b*^4^×*A*_0_)-(10*b*^3^×${A}_{1_{\mathrm{corr}}}$)-(10*b*^2^×${A}_{2_{\mathrm{corr}}}$)-(5*b*×${A}_{3_{\mathrm{corr}}}$)-[(16*c*×20*ab*)×*A*_0_]-[(16*c*×5*a*)×${A}_{1_{\mathrm{corr}}}$]-[(16*c*×10b^3^)×*A*_0_]-[(16*c*×10*b*^2^)×${A}_{1_{\mathrm{corr}}}$]-[(16*c*×5*b*)×${A}_{2_{\mathrm{corr}}}$]-(16*c*×${A}_{3_{\mathrm{corr}}}$)-[(120*c*^2^×5*a*)×*A*_0_]-[120*c*^2^×10*b*^2^)×*A*_0_]-[(120*c*^2^×5*b*)×${A}_{1_{\mathrm{corr}}}$]-(120*c*^2^×${A}_{2_{\mathrm{corr}}}$)-[(560*c*^3^×5*b*)×*A*_0_]-(560*c*^3^×${A}_{1_{\mathrm{corr}}}$)-(1820*c*^4^×*A*_0_)
${A}_{5_{\mathrm{corr}}}$	*A*_5_-(*b*^5^×*A*_0_)-(30*a*^2^*b*×*A*_0_)-(20*ab*×${A}_{2_{\mathrm{corr}}}$)-(20*ab*^3^×*A*_0_)-(30*ab*^2^×${A}_{1_{\mathrm{corr}}}$)-(10*a*^2^×${A}_{1_{\mathrm{corr}}}$)-(5*a*×${A}_{3_{\mathrm{corr}}}$)-(5*b*^4^×${A}_{1_{\mathrm{corr}}}$)-(10*b*^3^×${A}_{2_{\mathrm{corr}}}$)-(10*b*^2^×${A}_{3_{\mathrm{corr}}}$)-(5*b*×${A}_{4_{\mathrm{corr}}}$)-[(16*c*×20*ab*)×${A}_{1_{\mathrm{corr}}}$]-[(16*c*×30*ab*^2^)×*A*_0_]-[(16*c*×10*a*^2^)×*A*_0_]-[(16*c*×5*a*)×${A}_{2_{\mathrm{corr}}}$]-[(16*c*×5*b*^4^)×*A*_0_]-[(16*c*×10*b*^3^)×${A}_{1_{\mathrm{corr}}}$]-[(16*c*×10*b*^2^)×${A}_{2_{\mathrm{corr}}}$]-[(16*c*×5*b*)×${A}_{3_{\mathrm{corr}}}$]-(16*c*×${A}_{4_{\mathrm{corr}}}$)-[(120*c*^2^×20*ab*)×*A*_0_]-[(120*c*^2^×5*a*)×${A}_{1_{\mathrm{corr}}}$]-[(120*c*^2^×10*b*^3^)×*A*_0_]-[(120*c*^2^×10*b*^2^)×${A}_{1_{\mathrm{corr}}}$]-[(120*c*^2^×5*b*)×${A}_{2_{\mathrm{corr}}}$]-(120*c*^2^×${A}_{3_{\mathrm{corr}}}$)-[(560*c*^3^×5*a*)×*A*_0_]-[(560*c*^3^×10*b*^2^)×*A*_0_]-[(560*c*^3^×5*b*)×${A}_{1_{\mathrm{corr}}}$]-(560*c*^3^×${A}_{2_{\mathrm{corr}}}$)-[(1820*c*^4^×5*b*)×*A*_0_]-(1820*c*^4^×${A}_{1_{\mathrm{corr}}}$)-(4368*c*^5^×*A*_0_)

For the other metabolites used in the present study, the same approach, although obviously adjusted for the number of carbon atoms in the backbone as well as the elemental composition of the derivatization groups, was used. Expressions for naturally distributed isotope interference correction for metabolites used for the flux modeling (i.e., GAP, DHAP, E4P, Rl5P, R5P, F6P, and S7P) are given in Table S1.

**Fig. 2:**
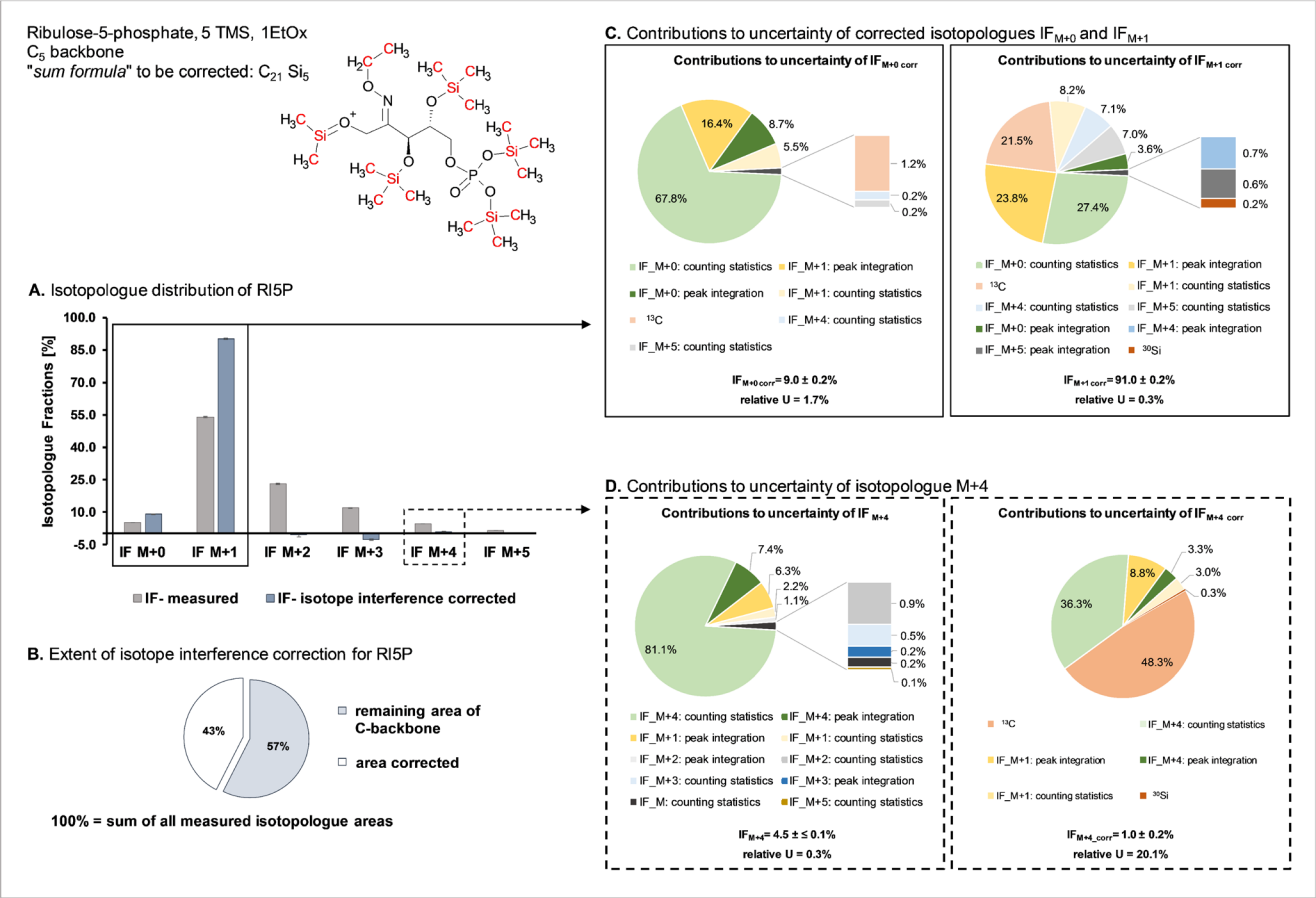
Contributions to the measurement uncertainty of selected isotopologue fractions (IFs) of ribulose 5-phosphate (Rl5P). Data were obtained from a metabolic flux experiment using [1,6-13C_2_]glucose as a tracer molecule. The depicted chemical structure of the fully derivatized Rl5P [M − CH_3_]^+^ ion highlights in red the naturally abundant elements introduced by derivatization, necessitating an interference correction. The bar graph in part A shows the isotopologue distributions obtained for the six isotopologues of Rl5P; the isotopologue distribution obtained by gas chromatography–chemical ionization time-of-flight mass spectrometry measurement [25] is shown in gray, whereas the IFs after isotope interference correction are depicted in blue. Part B illustrates the extent of naturally abundant isotope interference by a pie chart: if the sum of all measured isotopologue peak areas is considered to be 100%, 43% stem solely from derivatization groups. The pie charts in part C show the contributions to the uncertainty of IF_M+0corr_ and IF_M+1corr_, whereas in part D the pie charts indicate the contributions to uncertainty before (i.e., IF_M+4_) and after (i.e., IF_M+4corr_) isotope interference correction. EtOx ethoxime, TMS trimethylsilyl

In Fig. 2 part A the isotopologue distribution obtained from the aforementioned ^13^C-based MFA experiment [12] is shown for the sugar phosphate Rl5P. Elements being corrected for interferences (namely, naturally distributed silicon and carbon) are highlighted in red in the chemical structure depicted in the upper left part of Fig. 2. In the bar graph in Fig. 2 part A, the measured isotopologue distribution is shown in gray, whereas the isotopologue distribution after interference correction is shown in blue. As the data stem from an MFA experiment studying the PPP and glycolysis in *P. pastoris*, [1,6-^13^C_2_]glucose was used as a tracer [36], resulting in a predominant IF of M+1. As illustrated in Fig. 2 part B, considering the sum of all measured isotopologue peak areas as 100%, 43% of the area corresponds to naturally abundant heavy stable isotope interferences due to the presence of six derivatization groups.

It can be clearly seen in Fig. 2 part A that the interference-corrected IFs M+2 to M+5 show either low abundance or even negative values. The contribution of input quantities to the measurement uncertainty of the two remaining IFs (i.e., IF_M+1 corr_ and IF_M+0 corr_) is depicted in Fig. 2 part C. The uncertainty of the lower-abundance IF_M+0 corr_ is considerably higher than that of IF_M+1 corr_, and can be explained by the higher impact of counting statistics Besides, all IFs contribute at least to some extent to the uncertainty of IF_M+1 corr_ and IF_M+0 corr_. This can be explained by the fact that each fraction is calculated on the basis of the sum of all possible IFs.

After isotope interference correction, low-abundance values show a significant increase in uncertainty as shown exemplarily for IF_M+4_ in Fig. 2 part D. Before isotope interference correction, the major contribution to the standard uncertainty of IF_M+4_ is the counting statistics of the respective isotopologue. Additionally, the peak integration of IF_M+4_ and IF_M+1_ due to their high abundance contributes to the relative combined uncertainty of 0.3% obtained. After isotope interference correction, it can be seen that, apart from counting statistics, mainly the variance of the natural abundance of ^13^C affects the measurement uncertainty. This significant increase in measurement uncertainty (namely, from 0.3% to 20.1% relative combined uncertainty) leads to the consideration of discarding low-abundance IFs, as they show an inherent higher uncertainty, for the consecutive modeling part.

For the other metabolites (namely, GAP, DHAP, E4P, R5P, F6P, G6P, and S7P) full data on measured isotopologues as well as isotope-interference-corrected fractions, including the respective uncertainties, are given in Table S2.

## Impact of measurement uncertainty on the estimation of metabolic fluxes

**Fig. 3:**
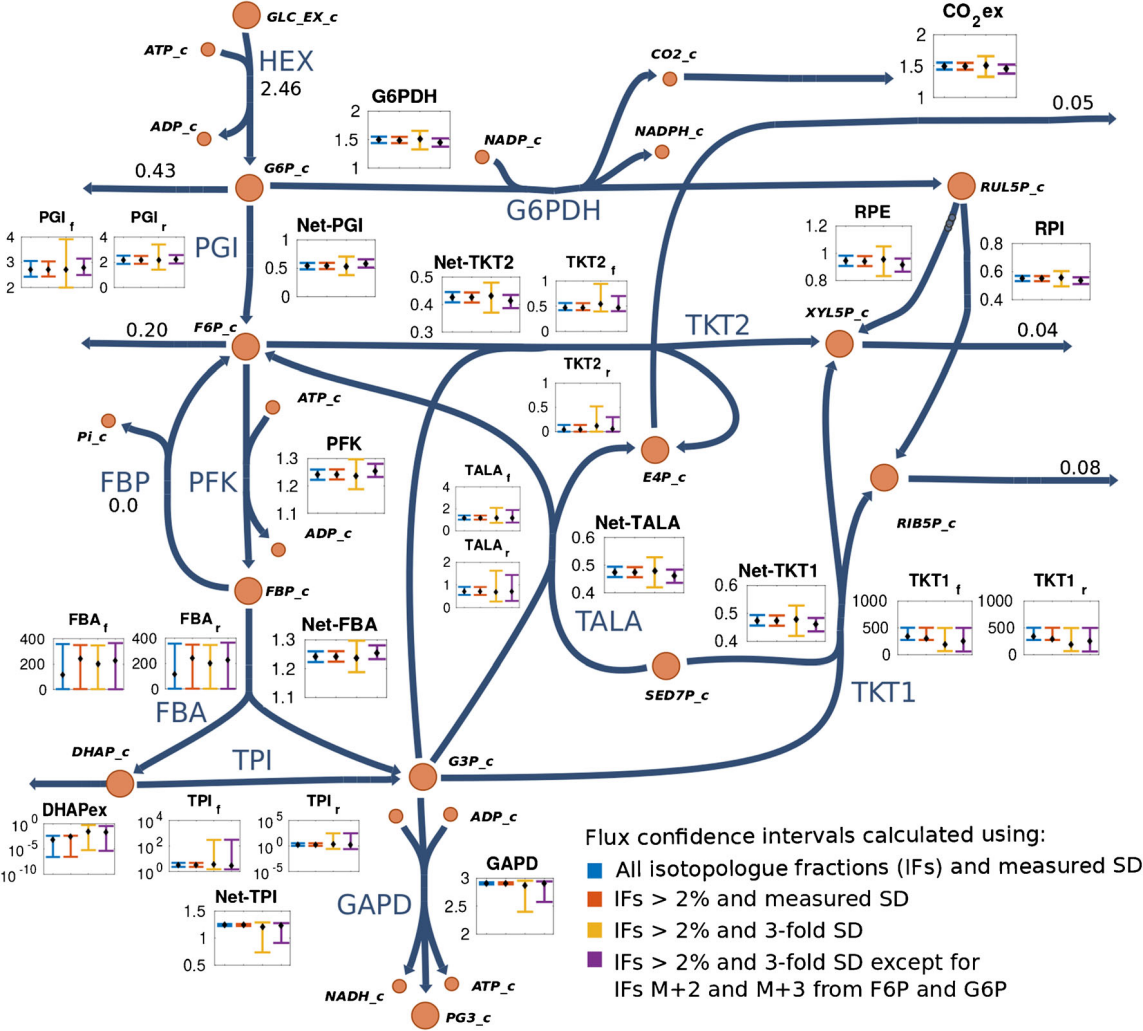
Effect of isotopologue fraction (IF) uncertainties on flux confidence intervals. Estimated fluxes (dots) along with their confidence intervals (bars) in different scenarios are depicted in the small figures associated with each reaction. Removal of IFs with an abundance of less than 2% has no effect on confidence intervals (compare the blue and red bars). Flux estimation, when the biological variance (three times the standard deviation) was included, yields threefold larger confidence intervals (yellow bars). Increasing the precision of critical analytes [IFs M+1and M+2 from fructose 6-phosphate (F6P) and glucose 6-phosphate (G6P)] has the largest impact on reducing flux uncertainty (purple bars). For reversible reactions, forward, reverse, and net fluxes are shown. Fluxes are given in millimoles per gram dry cell weight per hour. SD standard deviation. ADP adenosine diphosphate, ATP adenosine triphosphate, DHAP dihydroxyacetone phosphate, E4P erythrose 4-phosphate, FBA fructose bisphosphate aldolase, FBP fructose 1,6-bisphosphate, GAPDH glyceraldehyde 3-phosphate dehydrogenase, GLC glucose, G3P glyceraldehyde 3-phosphate, G6PDH glucose 6-phosphate dehydrogenase, HEX hexokinase, NADP nicotinamide adenine dinucleotide phosphate, NADPH reduced nicotinamide adenine dinucleotide phosphate, PI phosphate, PFK phosphofructokinase, PG3 3-phosphoglyceric acid, PGI glucose 6-phosphate isomerase, RIB5P ribose 5-phosphate, RPE ribulose 5-phosphate epimerase, RPI ribose 5-phosphate isomerase, RUL5P ribulose 5-phosphate, SED7P sedoheptulose 7-phosphate , TALA transaldolase, TKT1 transketolase 1, TKT2 transketolase 2, TPI triosephosphate isomerase, XYL5P xylulose-5-phosphate

**Fig. 4:**
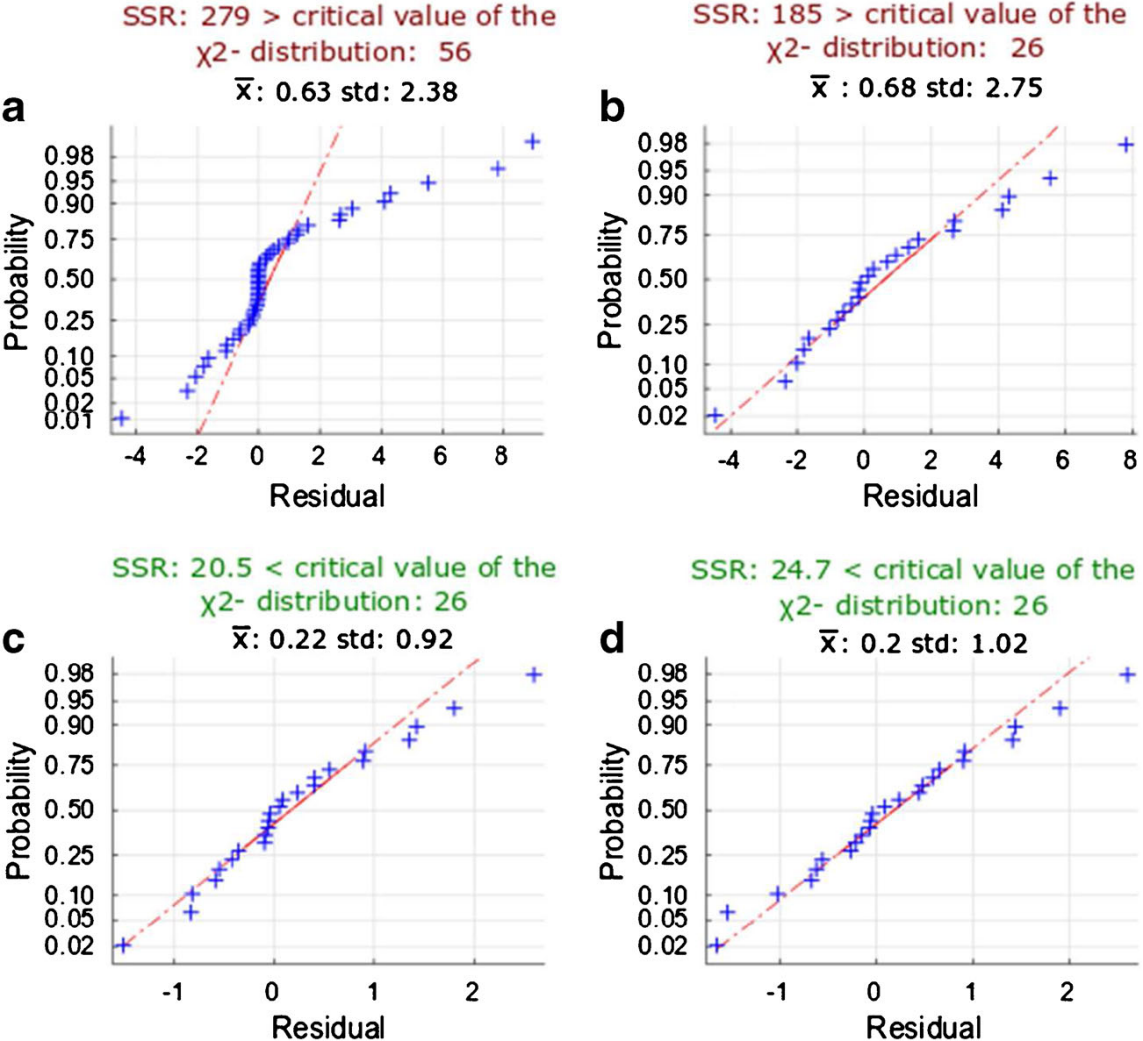
Normal probability plots for the standard-deviation-weighted residuals after flux estimation. Deviation of a normal distribution (=0, =1) indicates errors in the metabolic model or in the measurement uncertainties of isotopologue fractions (IFs). Fluxes were estimated with use of **a** all IFs with their modeled standard uncertainty, **b** only IFs with abundance greater than 2%, **c** only IFs with abundance greater than 2% with three times their standard uncertainty to account for biological variability, and **d** only IFs with abundance greater than 2% with three times their standard deviation except for the isotopologues M+1 and M+2 from fructose 6-phosphate and glucose 6-phosphate. SSR sum of squared residuals

As demonstrated in the previous section, low-abundance IFs result in high relative uncertainty. Thus, it is interesting to investigate the impact of these small fractions on the flux estimation. For this purpose, confidence intervals were calculated with use of only IFs with abundance greater than 2%, as well as with the complete set of isotope-interference-corrected IFs. As observed in Fig. 3, the resulting confidence intervals are essentially identical, which indicates that small IFs have a negligible effect on fluxes. Given that [1,6-^13^C_2_]glucose was used as tracer in the experiment, isotopologues with a higher mass increment were not expected to be generated for the reactions that were the focus of the present study (e.g., IFs of R5P from M+2 to M+5; see Fig. 2 part A). These IFs lead artificially to an improper weighting of the residuals and could bias parameter estimation during the subsequent fitting procedure [27]. The latter was observed from the improved sum of squared residuals that is obtained after removal of the small IFs, as well as from the normal probability plot of the standard-deviation-weighted residuals (compare Fig. 4a and b). Also, removal of IFs smaller than 2% shifted the distribution of weighted residuals toward the expected normal distribution (Fig. 4b), which then passed a Shapiro–Wilk test for normal distribution. However, the mean and standard deviation of this distribution still deviated from the expected values of 0 and 1, respectively. Moreover, statistical tests on the goodness of fit revealed bad fitting, since the variance-weighted sum of squared residuals did not pass the critical value of the X^2^ distribution for a confidence level of 95%.

Thus, at this point of the workflow, the main factors influencing the uncertainty of metabolic fluxes are the quality of the metabolic model and the accuracy of measured IFs, rather than their precision, which is on average 2%. Regarding the metabolic model, several reactions (e.g*.*, trehalose 6-phosphate synthase) are not included or are possibly not annotated [37, 38], leading to an unaccounted influence on the labeling patterns. Comparison of the IFs measured for 6-phosphogluconate and G6P supports this idea (Table S3). Even though these two compounds are expected to be part of an irreversible linear pathway, significant differences in their labeling patterns were observed. Thus, unaccounted reactions may significantly shift the ^13^C labeling patterns. As for the accuracy of IFs, with increasing precision of the analytical procedure the investigation of potential biases on the true value becomes increasingly important. The analytical approach presented satisfies this requirement of delivering highly precise values; however, it has to be emphasized that biases introduced by the analytical method cannot be corrected currently because of the lack of suitable reference standards. Another reason for metabolite-specific bias might be differences in the cell populations (e.g., cell cycle stage, age) in the batch culture used to generate the data. This could be especially relevant for analytes in low concentrations in the cell and/or that are affected by low derivatization efficiency (e.g., E4P).

**Fig. 5:**
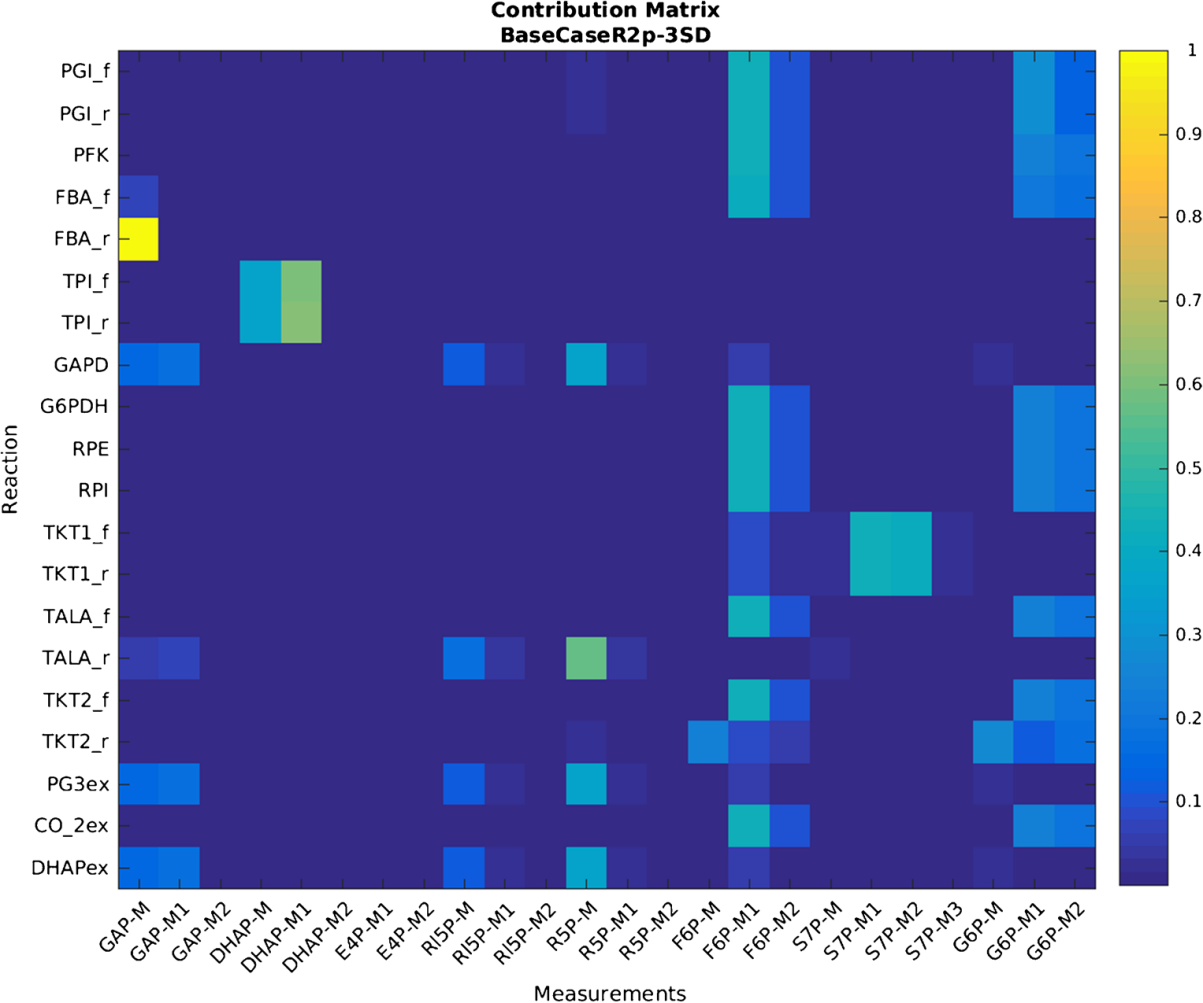
Contribution matrix of isotopologue analysis with regard to flux precision. Uncertainty of isotopologue fractions (IFs) M+1 and M+2 from fructose 6-phosphate (F6P) and glucose 6-phosphate (G6P) has the largest impact on the precision of most of the fluxes, as indicated by the color plot on the right *y*-axis. ADP adenosine diphosphate, ATP adenosine triphosphate, DHAP dihydroxyacetone phosphate, E4P erythrose 4-phosphate, FBA fructose bisphosphate aldolase, FBP fructose 1,6-bisphosphate, GAPDH glyceraldehyde 3-phosphate dehydrogenase, GLC glucose, G3P glyceraldehyde 3-phosphate, G6PDH glucose 6-phosphate dehydrogenase, HEX hexokinase, NADP nicotinamide adenine dinucleotide phosphate, NADPH reduced nicotinamide adenine dinucleotide phosphate, PI phosphate, PFK phosphofructokinase, PG3 3-phosphoglyceric acid, PGI glucose 6-phosphate isomerase, RIB5P ribose 5-phosphate, RPE ribulose 5-phosphate epimerase, RPI ribose 5-phosphate isomerase, RUL5P ribulose 5-phosphate, SED7P sedoheptulose 7-phosphate , TALA transaldolase, TKT1 transketolase 1, TKT2 transketolase 2, TPI triosephosphate isomerase, XYL5P xylulose-5-phosphate

Assuming that the model cannot be further improved and data accuracy cannot be further increased, one can obtain a statistically significant flux estimation by accounting also for the biological variance stemming from the batch culture of unsynchronized cells by extending the standard deviation originating from the analytical procedure with an estimated factor of 3. However, any increase in the standard uncertainty of key measurements directly correlates with an increase in the uncertainty of fluxes [27]. With use of this biological factor of 3 for the distribution of the IFs, parameter estimation passed a test for goodness of fit, but the size of the confidence intervals for the reactions of interest (i*.e.*, glucose 6-phosphate dehydrogenase (G6PDH) and the net flux through phosphoglucose isomerase (PGI)) increased almost threefold as well (Fig. 3, yellow bars). In addition, forward and reverse fluxes of the triose phosphate isomerase reaction became unidentifiable. Notably, only certain measurements contribute significantly to the estimation of each flux, as described by the contribution matrix [27]. Computation of the contribution matrix (Fig. 5) revealed that the measurements of IFs M+1 and M+2 of F6P and G6P have the largest contribution to the flux estimation of the glucose 6-phosphate dehydrogenase and phosphoglucose isomerase reactions. To illustrate the importance of these contributing measurements, fluxes were recalculated with the measured standard deviations of the three biological replicates [25] for these isotopologues and an estimated biological factor for 3 for all others. This resulted in a statistically significant flux fitting with increased precision for the fluxes of several reactions, in particular the reactions of interest; namely, glucose 6-phosphate dehydrogenase and the net flux through phosphoglucose isomerase (see Fig. 3). These findings clearly indicate that the model of the investigated branching point is highly robust. Full data on measured and simulated IFs obtained with the strategies discussed for handling uncertainties in the simulation process are given in Table S4.

As recently reported by Theorell et al. [39], uncertainty quantification in the form of confidence intervals calculated by Monte Carlo simulation yields a rather optimistic estimation of flux uncertainty. However, this method was selected for our analysis for its simple implementation (including the consideration of covariances) and robustness and, even more importantly, because of its implementation in internationally accepted guidelines such as in the *Guide to the Expression of Uncertainty in Measurement* guidelines [23]. Similarly, the X^2^ test for goodness of fit is recommended as standard practice in ^13^C-based MFA [40]. However, it depends heavily on a correct estimation of measurement uncertainty given by the covariance matrix [39]. This increases the need for curation of the metabolic model structure (e.g., by enzymatic assays or tailored ^13^C labeling experiments) [41]. Given a correct model structure, it is thus possible to use the X^2^ test to guide the modeling of measurement uncertainty.

## Conclusion

Although the isotopologue distribution of free intracellular metabolites is determined with high precision, the true value of IFs within a ^13^C-based MFA experiment remains poorly characterized because of the lack of a suitable certified matrix reference material for isotopologue analysis. The value of such a reference material was recently demonstrated by Heuillet et al. [42]. Besides, our analysis also pointed out the underlying metabolic model as a structural source of error, as also suggested in other studies [40, 41]. Thus, substantial efforts should focus on improved model curation by capturing all reactions affecting measured metabolites. As a further conclusion, we recommend a priori identification of metabolites involved in the metabolic fluxes of interest and to specifically focus on these with, for example, dedicated preconcentration steps for certain low-abundance metabolites and thereby potentially increase accuracy.

## Electronic supplementary material

ESM 1 (PDF 636 kb)

ESM 2 (XLSX 23910 kb)

ESM 3 (XLS 40 kb)
