# Supplementary material for: Comprehensive assessment of measurement uncertainty in 13C-based metabolic flux experiments
Source: Anal Bioanal Chem. 2018 Apr 13;410(14):3337–48. doi: 10.1007/s00216-018-1017-7 (PMC5937919; doi:10.1007/s00216-018-1017-7)
Supplement: Supplementary file 1 — (PDF 636 kb) [file 216_2018_1017_MOESM1_ESM.pdf]

**Analytical and Bioanalytical Chemistry**

**Electronic Supplementary Material**

# Comprehensive assessment of measurement uncertainty in $^{13}$C based metabolic flux experiments

Teresa Mairinger, Wolfhard Wegscheider, David Alejandro Peña, Matthias G. Steiger, Gunda Koellensperger, Jürgen Zanghellini, Stephan Hann

Additional files available under 10.1007/s00216-018-1017-7

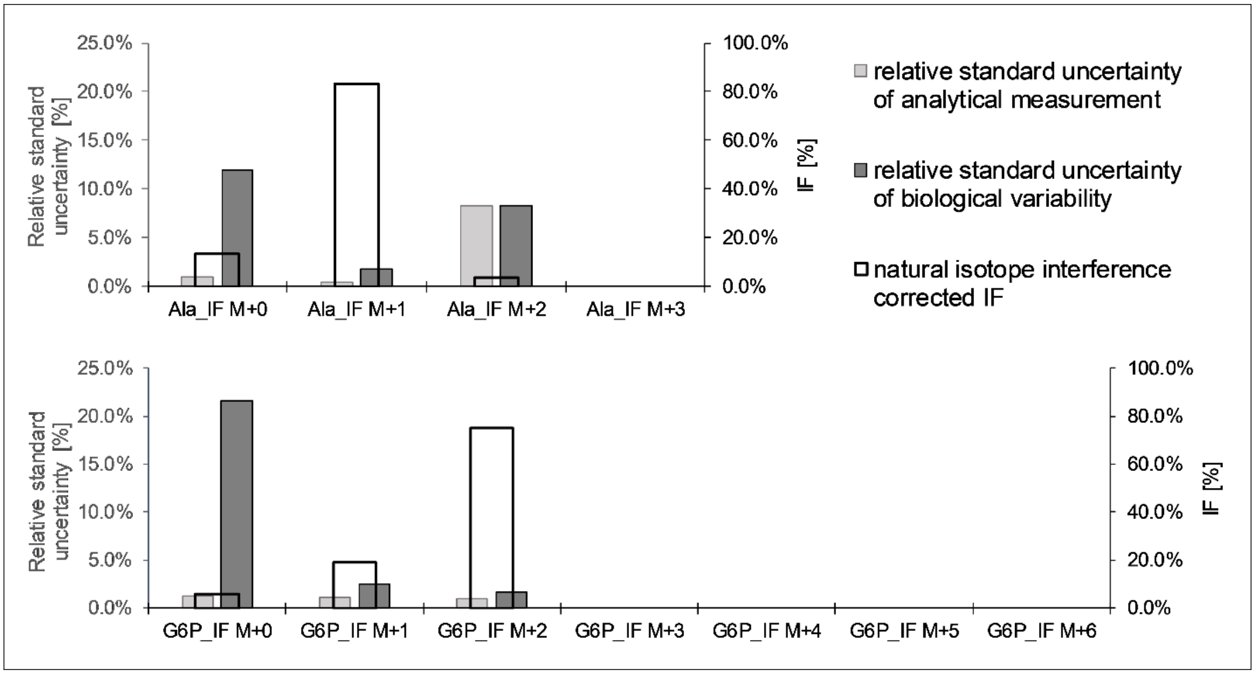


**Fig. S1** Exemplary comparison of relative standard uncertainties originating either from the analytical measurement procedure or from the biological variability from glucose-6-Phosphate (G6P) and alanine (Ala). Apart from others, biological variability can be explained by differences in terms homogeneity of the studied cell population, environmental factors as well as metabolite specific turnover rates, making optimal quenching and sampling conditions central. For assessing the biological variability, the following model equation was used: $IF_{M+n\ corr} * f_{bio}$ ($f_{bio}$= biological factor (distribution = normal, $\bar{x}$= 1, s= specific)). For this purpose, the standard deviation of three biological replicates was assessed, subsequently corrected for the analytical uncertainty and applied as the variation s for the biological factor. To illustrate the dependence on the isotopologue fraction (IF)

**Table S1** Equations for isotope interference correction of alanine (Ala) phosphoenolpyruvate (PEP), glyceraldehyde 3-phosphate (GAP), dihydroxyacetone phosphate (DHAP), 2-Phosphoglycerat (2PG), 3-Phosphoglycerat (3PG), erythrose-4-phosphate (E4P), ribulose-5-phosphate (Rl5P), ribose-5-phosphate (R5P), glucose-6-phosphate (G6P), fructose-6-phosphate (F6P) and seduheptulose-7-phosphate (S7P) are given

**Ala:** $C_3$-backbone, to be corrected *sum formula*: $C_9$ $Si_2$

| **isotope inference corrected area of M+ *n* isotopologue** | **Equation for correction** |
|---|---|
| $A_0$ | $A_0$ |
| $A_{1_{corr}}$ | $A_1$-(2b*$A_0$)-(6c*$A_0$) |
| $A_{2_{corr}}$ | $A_2$-($b^2$*$A_0$)-(2a*$A_0$)-(2b*$A_{1_{corr}}$)-((6c*2b)*$A_0$)-(6c*$A_{1_{corr}}$)-(15$c^2$*$A_0$) |
| $A_{3_{corr}}$ | $A_3$-($b^2$*$A_{1_{corr}}$)-(2ab*$A_0$)-(2a*$A_{1_{corr}}$)-(2b*$A_2$)-((6c*$b^2$)*$A_0$)-((6c*2a)*$A_0$)-((6c*2b)* $A_{1_{corr}}$)-(6c*$A_{2_{corr}}$)-((15$c^2$*2b)*$A_0$)-(15$c^2$* $A_{1_{corr}}$)-(20$c^3$*$A_0$) |

**PEP:** $C_3$-backbone, to be corrected *sum formula*: $C_{12}$ $Si_3$

| **isotope inference corrected area of M+ *n* isotopologue** | **Equation for correction** |
|---|---|
| $A_0$ | $A_0$ |
| $A_{1_{corr}}$ | $A_1$-(3b*$A_0$)-(9c-$A_0$) |
| $A_{2_{corr}}$ | $A_2$-(3$b^2$*$A_0$)-(3a-$A_0$)-(3b-$A_{1_{corr}}$)-((9c*3b)*$A_0$)-(9c*$A_{1_{corr}}$)-(36$c^2$*$A_0$) |
| $A_{3_{corr}}$ | $A_3$-($b^3$*$A_0$)-(6ab*$A_0$)-(3$b^2$*$A_{1_{corr}}$)-(3a*$A_{1_{corr}}$)-(3b*$A_{2_{corr}}$)-((9c*3$b^2$)*$A_0$)-((9c*3a)*$A_0$)-((9c*3b)* $A_{1_{corr}}$)-(9c*$A_{2_{corr}}$)-((36$c^2$*3b)*$A_0$)-(36$c^2$*$A_{1_{corr}}$)-(84$c^3$*$A_0$) |

**GAP/DHAP:** $C_3$-backbone, to be corrected *sum formula* $C_{14}$ $Si_3$

| **isotope inference corrected area of M+ *n* isotopologue** | **Equation for correction** |
|---|---|
| $A_0$ | $A_0$ |
| $A_{1_{corr}}$ | $A_1$-(3b*$A_0$)-(11c-$A_0$) |
| $A_{2_{corr}}$ | $A_2$-(3$b^2$*$A_0$)-(3a*$A_0$)-(3b*$A_{1_{corr}}$)-((11c*3b)*$A_0$)-(11c*$A_{1_{corr}}$)-(55$c^2$*$A_0$) |
| $A_{3_{corr}}$ | $A_3$-($b^3$*$A_0$)-(6ab*$A_0$)-(3$b^2$*$A_{1_{corr}}$)-(3a*$A_{1_{corr}}$)-(3b*$A_{2_{corr}}$)-((11c*3$b^2$)*$A_0$)-((11c*3a)*$A_0$)-((11c*3b)* $A_{1_{corr}}$)-(11c*$A_{2_{corr}}$)-((55$c^2$*3b)*$A_0$)-(55$c^2$*$A_{1_{corr}}$)-(165$c^3$*$A_0$) |

**2PG/3PG:** $C_3$-backbone, to be corrected *sum formula*: $C_{15}$ $Si_4$

| **isotope inference corrected area of M+ *n* isotopologue** | **Equation for correction** |
|---|---|
| $A_0$ | $A_0$ |
| $A_{1_{corr}}$ | $A_1-(4b*A_0)-(12c*A_0)$ |
| $A_{2_{corr}}$ | $A_2-(4a*A_0)-(6b^2*A_0)-(4b*A_{1_{corr}})-((12c*4b)*A_0)-(12c*A_{1_{corr}})-(66c^2*A_0)$ |
| $A_{3_{corr}}$ | $A_3-(12ab*A_0)-(4a*A_{1_{corr}})-(4b^3*A_0)-(6b^2*A_{1_{corr}})-(4b*A_{2_{corr}})-((12c*4a)*A_0)-((12c*6b^2)*A_0)-$<br>$((12c*4b)*A_{1_{corr}})-(12c*A_{2_{corr}})-(66c^2*4b)*A_0)-(66c^2*A_{1_{corr}})-(220c^3*A_0)$ |

**E4P:** $C_4$-backbone, to be corrected *sum formula*: $C_{17}$ $Si_4$

| **isotope inference corrected area of M+ *n* isotopologue** | **Equation for correction** |
|---|---|
| $A_0$ | $A_0$ |
| $A_{1_{corr}}$ | $A_1-(4b*A_0)-(13c*A_0)$ |
| $A_{2_{corr}}$ | $A_2-(4a*A_0)-(6b^2*A_0)-(4b*A_{1_{corr}})-((13c*4b)*A_0)-(13c*A_{1_{corr}})-(78c^2*A_0)$ |
| $A_{3_{corr}}$ | $A_3-(12ab*A_0)-(4a*A_{1_{corr}})-(4b^3*A_0)-(6b^2*A_{1_{corr}})-(4b*A_{2_{corr}})-((13c*4a)*A_0)-((13c*6b2)*A_0)-$<br>$((13c*4b)*A_{1_{corr}})-(13c*A_{2_{corr}})-((78c^2*4b)*A_0)-(78c^2*A_{1_{corr}})-(286c^3*A_0)$ |
| $A_{4_{corr}}$ | $A_4-(b^4*A_0)-(12ab*A_{1_{corr}})-(12ab^2*A_0)-(6a^2*A_0)-(4a*A_{2_{corr}})-(4b^3-A_{1_{corr}})-(6b^2*A_{2_{corr}})-(4b-A_{3_{corr}})-$<br>$((13c*12ab)*A_0)-((13c*4a)*A_{1_{corr}})-((13c*4b^3)*A_0)-((13c*6b^2)*A_{1_{corr}})-((13c*4b)*A_{2_{corr}})-$<br>$(13c*A_{3_{corr}})-((78c^2*4a)*A_0)-((78c^2*6b^2)*A_0)-((78c^2*4b)*A_{1_{corr}})-(78c^2*A_{2_{corr}})-((286c^3*4b)*A_0)-$<br>$(286c^3*A_{1_{corr}})-(715c^4*A_0)$ |

**R5P/RI5P:** $C_5$-backbone, to be corrected *sum formula*: $C_{21}\ Si_5$

| **isotope inference corrected area of M+ *n* isotopologue** | **Equation for correction** |
|---|---|
| $A_0$ | $A_0$ |
| $A_{1_{corr}}$ | $A_1-(5b*A_0)-(16c*A_0)$ |
| $A_{2_{corr}}$ | $A_2-(5a*A_0)-(10b^2*A_0)-(5b*A_{1_{corr}})-((16c*5b)*A_0)-(16c*A_{1_{corr}})-(120c^2*A_0)$ |
| $A_{3_{corr}}$ | $A_3-(20ab*A_0)-(5a*A_{1_{corr}})-(10b^3*A_0)-(10b^2*A_{1_{corr}})-(5b*A_{2_{corr}})-((16c*5a)*A_0)-((16c*10b^2)*A_0)-((16c*5b)*A_{1_{corr}})-(16c*A_{2_{corr}})-((120c^2*5b)*A_0)-(120c^2*A_{1_{corr}})-(560c^3*A_0)$ |
| $A_{4_{corr}}$ | $A_4-(20ab*A_{1_{corr}})-(30ab^2*A_0)-(10a^2*A_0)-(5a*A_{2_{corr}})-(5b^4*A_0)-(10b^3*A_{1_{corr}})-(10b^2*A_{2_{corr}})-(5b*A_{3_{corr}})-((16c*20ab)*A_0)-((16c*5a)*A_{1_{corr}})-((16c*10b^3)*A_0)-((16c*10b^2)*A_{1_{corr}})-((16c*5b)*A_{2_{corr}})-(16c*A_{3_{corr}})-((120c^2*5a)*A_0)-(120c^2*10b^2)*A_0)-((120c^2*5b)*A_{1_{corr}})-(120c^2*A_{2_{corr}})-((560c^3*5b)*A_0)-(560c^3*A_{1_{corr}})-(1820c^4*A_0)$ |
| $A_{5_{corr}}$ | $A_5-(b5*A_0)-(30a^2b*A_0)-(20ab*A_{2_{corr}})-(20ab^3*A_0)-(30ab^2*A_{1_{corr}})-(10a^2*A_{1_{corr}})-(5a*A_{3_{corr}})-(5b^4*A_{1_{corr}})-(10b^3*A_{2_{corr}})-(10b^2*A_{3_{corr}})-(5b*A_{4_{corr}})-((16c*20ab)*A_{1_{corr}})-((16c*30ab^2)*A_0)-((16c*10a^2)*A_0)-((16c*5a)*A_{2_{corr}})-((16c*5b^4)*A_0)-((16c*10b^3)*A_{1_{corr}})-((16c*10b^2)*A_{2_{corr}})-((16c*5b)*A_{3_{corr}})-(16c*A_{4_{corr}})-((120c^2*20ab)*A_0)-((120c^2*5a)*A_{1_{corr}})-((120c^2*10b^3)*A_0)-((120c^2*10b^2)*A_{1_{corr}})-((120c^2*5b)*A_{2_{corr}})-(120c^2*A_{3_{corr}})-((560c^3*5a)*A_0)-((560c^3*10b^2)*A_0)-((560c^3*5b)*A_{1_{corr}})-(560c^3*A_{2_{corr}})-((1820c^4*5b)*A_0)-(1820c^4*A_{1_{corr}})-(4368c^5*A_0)$ |

**F6P/G6P:** $C_6$-backbone, to be corrected *sum formula*: $C_{25}\ Si_6$

| **isotope inference corrected area of M+ *n* isotopologue** | **Equation for correction** |
|---|---|
| $A_0$ | $A_0$ |
| $A_{1_{corr}}$ | $A_1-(6b*A_0)-(19c*A_0)$ |
| $A_{2_{corr}}$ | $A_2-(6a*A_0)-(15b^2*A_0)-(6b*A_{1_{corr}})-((19c*6b)*A_0)-(19c*A_{1_{corr}})-(171c^2*A_0)$ |
| $A_{3_{corr}}$ | $A_3-(30ab*A_0)-(6a*A_{1_{corr}})-(20b^3*A_0)-(15b^2*A_{1_{corr}})-(6b*A_{2_{corr}})-((19c*6a)*A_0)-((19c*15b^2)*A_0)-((19c*6b)* A_{1_{corr}})-(19c*A_{2_{corr}})-((6b*171c^2)*A_0)-(171c^2*A_{1_{corr}})*(969c^3*A_0)$ |
| $A_{4_{corr}}$ | $A_4-(30ab*A_{1_{corr}})-(60ab^2*A_0)-(15a^2*A_0)-(6a*A_{2_{corr}})-(15b^4*A_0)-(20b^3*A_{1_{corr}})-(15b^2*A_{2_{corr}})-(6b*A_{3_{corr}})-((19c*30ab)*A_0)-((19c*6a)* A_{1_{corr}})-((19c*20b^3)*A_0)-((19c*15b^2)* A_{1_{corr}})-((19c*6b)* A_{2_{corr}})-(19c*A_{3_{corr}})-((171c^2*6a)*A_0)-((171c^2*15b^2)*A_0)-((171c^2*6b)* A_{1_{corr}})-(171c^2*A_{2_{corr}})-((969c^3*6b)*A_0)-(969c^3*A_{1_{corr}})-(3876c^4*A_0)$ |
| $A_{5_{corr}}$ | $A_5-(60a^2b*A_0)-(30ab*A_{2_{corr}})-(60ab^3*A_0)-(60ab^2*A_{1_{corr}})-(15a^2*A_{1_{corr}})-(6a*A_{3_{corr}})-(6b^5*A_0)-(15b^4*A_{1_{corr}})-(20b^3*A_{2_{corr}})-(15b^2*A_{3_{corr}})-(6b*A_{4_{corr}})-((19c*30ab)* A_{1_{corr}})-((19c*60ab^2)*A_0)-((19c*15a^2)*A_0)-((19c*6a)* A_{2_{corr}})-((19c*15b^4)*A_0)-((19c*20b^3)* A_{1_{corr}})-((19c*15b^2)* A_{2_{corr}})-((19c*6b)* A_{3_{corr}})-(19c*A_{4_{corr}})-((171c^2*30ab)*A_0)-((171c^2*6a)* A_{1_{corr}})-((171c^2*20b^3)*A_0)-((171c^2*15b^2)* A_{1_{corr}})-((171c^2*6b)* A_{2_{corr}})-(171c^2*A_{3_{corr}})-((969c^3*6a)*A_0)-((969c^3*15b^2)*A_0)-((969c^3*6b)* A_{1_{corr}})-(969c^3*A_{2_{corr}})-((3876c^4*6b)*A_0)-(3876c^4*A_{1_{corr}})-(11628c^5*A_0)$ |
| $A_{6_{corr}}$ | $A_6-(b^6*A_0)-(60a^2b*A_{1_{corr}})-(30ab*A_{3_{corr}})-(30ab^4*A_0)-(60ab^3*A_{1_{corr}})-(60ab^2*A_{2_{corr}})-(90a^2b^2*A_0)-(20a^3*A_0)-(15a^2*A_{2_{corr}})-(6a*A_{4_{corr}})-(6b^5*A_{1_{corr}})-(15b^4*A_{2_{corr}})-(20b^3*A_{3_{corr}})-(15b^2*A_{4_{corr}})-(6b*A_{5_{corr}})-((19c*60a^2b)*A_0)-((19c*30ab)* A_{2_{corr}})-((19c*60ab^3)*A_0)-((19c*60ab^2)* A_{1_{corr}})-((19c*15a^2)* A_{1_{corr}})-((19c*6a)* A_{3_{corr}})-((19c*6b^5)*A_0)-((19c*15b^4)* A_{1_{corr}})-((19c*20b^3)* A_{2_{corr}})-((19c*15b^2)* A_{3_{corr}})-((19c*6b)* A_{4_{corr}})-(19c*A_{5_{corr}})-((171c^2*30ab)* A_{1_{corr}})-((171c^2*60ab^2)*A_0)-((171c^2*15a^2)*A_0)-((171c^2*6a)* A_{2_{corr}})-((171c^2*15b^4)*A_0)-(171c^2*20b^3)* A_{1_{corr}})-((171c^2*15b^2)* A_{2_{corr}})-((171c^2*6b)* A_{3_{corr}})-(171c^2*A_{4_{corr}})-((969c^3*30ab)*A_0)-((969c^3*6a)* A_{1_{corr}})-((969c^3*20b^3)*A_0)-((969c^3*15b^2)* A_{1_{corr}})-((969c^3*6b)* A_{2_{corr}})-(969c^3*A_{3_{corr}})-((3876c^4*6a)*A_0)-((3876c^4*15b^2)*A_0)-((3876c^4*6b)* A_{1_{corr}})-(3876c^4*A_{2_{corr}})-((11628c^5*6b)A_0)-(11628c^5*A_{1_{corr}})-(27132c^6*A_0)$ |

**S7P:** $C_7$-backbone, to be corrected *sum formula*: $C_{29}\,Si_7$

| **isotope inference corrected area of M+ *n* isotopologue** | **Equation for correction** |
|---|---|
| $A_0$ | $A_0$ |
| $A_{1_{corr}}$ | $A_1-(7b*A_0)-(22c*A_0)$ |
| $A_{2_{corr}}$ | $A_2-(7a*A_{1_{corr}})-(21b^2*A_0)-(7b*A_{1_{corr}})-((22c*7b)*A_0)-(22c*A_{1_{corr}})-(231c^2*A_0)$ |
| $A_{3_{corr}}$ | $A_3-(42ab*A_0)-(7a*A_{1_{corr}})-(35b^3*A_0)-(21b^2*A_{1_{corr}})-(7b*A_{2_{corr}})-((22c*7a)*A_0)-((22c*21b^2)*A_0)-((22c*7b)* A_{1_{corr}})-(22c*A_{2_{corr}})-((231c^2*7b)*A_0)-((231c^2*A_{1_{corr}})-(1540c^3*A_0)$ |
| $A_{4_{corr}}$ | $A_4-(42ab*A_{1_{corr}})-(105ab^2*A_0)-(21a^2*A_0)-(7a*A_{2_{corr}})-(35b^4*A_0)-(35b^3*A_{1_{corr}})-(21b^2*A_{2_{corr}})-(7b*A_{3_{corr}})-((22c*42ab)*A_0)-((22c*7a)* A_{1_{corr}})-((22c*35b^3)*A_0)-((22c*21b^2)* A_{1_{corr}})-((22c*7b)* A_{2_{corr}})-(22c*A_{3_{corr}})-((231c^2*7a)*A_0)-(( 231c^2*21b^2)*A_0)-( 231c^2*7b)* A_{1_{corr}})-(231c^2*A_{2_{corr}})-(1540c^3*7b)*A_0)-(1540c^3*A_{1_{corr}})-(7315c^4*A_0)$ |
| $A_{5_{corr}}$ | $A_5-(105a^2b*A_0)-(42ab*A_{2_{corr}})-(140ab^3*A_0)-(105ab^2*A_{1_{corr}})-(21a^2*A_{1_{corr}})-(7a*A_{3_{corr}})-(21b^5*A_0)-(35b^4*A_{1_{corr}})-(35b^3*A_{2_{corr}})-(21b^2*A_{3_{corr}})-(7b*A_{4_{corr}})-((22c*42ab)* A_{1_{corr}})-((22c*105ab^2)*A_0)-((22c*21a^2)*A_0)-((22c*7a)* A_{2_{corr}})-((22c*35c^4)*A_0)-((22c*35b^3)* A_{1_{corr}})-(22c*21b^2)* A_{2_{corr}})-((22c*7b)* A_{3_{corr}})-(22c*A_{4_{corr}})-((231c^2*42ab)*A_0)-((231c^2*7a)* A_{1_{corr}})-((231c^2*35b^3)*A_0)-((231c^2*21b^2)* A_{1_{corr}})-((231c^2*7b)* A_{2_{corr}})-(231c^2*A_{3_{corr}})-((1540c^3*7a)*A_0)-((1540c^3*21b^2)*A_0)-((1540c^3*7b)* A_{1_{corr}})-(1540c^3*A_{2_{corr}})-((7315c^4*7b)*A_0)-(7315c^4*A_{1_{corr}})-(26334c^5*A_0)$ |
| $A_{6_{corr}}$ | $A_6-(105a^2b*A_{1_{corr}})-(42ab*A_{3_{corr}})-(105ab^4*A_0)-(140ab^3*A_{1_{corr}})-(105ab^2*A_{2_{corr}})-(210a^2b^2*A_0)-(35a^3*A_0)-(21a^2*A_{2_{corr}})-(7a*A_{4_{corr}})-(7b^6*A_0)-(21b^5*A_{1_{corr}})-(35b^4*A_{2_{corr}})-(35b^3*A_{3_{corr}})-(21b^2*A_{4_{corr}})-(7b*A_{5_{corr}})-((22c*105a^2b)*A_0)-((22c*42ab)* A_{2_{corr}})-((22c*140ab^3)*A_0)-((22c*105ab^2)* A_{1_{corr}})-((22c*21a^2)* A_{1_{corr}})-((22c*7a)* A_{3_{corr}})-((22c*21b^5)*A_0)-((22c*35b^4)* A_{1_{corr}})-((22c*35b^3)* A_{2_{corr}})-((22c*21b^2)* A_{3_{corr}})-((22c*7b)* A_{4_{corr}})-(22c*A_{5_{corr}})-((231c^2*42ab)* A_{1_{corr}})-((231c^2*105ab^2)*A_0)-(231c^2*21a^2)*A_0)-(231c^2*7a)* A_{2_{corr}})-((231c^2*35b^4)*A_0)-((231c^2*35b^3)* A_{1_{corr}})-((231c^2*21b^2)* A_{2_{corr}})-((231c^2*7b)* A_{3_{corr}})-(231c^2*A_{4_{corr}})-((1540c^3*42ab)*A_0)-((1540c^3*7a)* A_{1_{corr}})-((1540c^3*35b^3)*A_0)-((1540c^3*21b^2)* A_{1_{corr}})-((1540c^3*7b)* A_{2_{corr}})-(1540c^3*A_{3_{corr}})-((7315c^4*7a)*A_0)-((7315c^4*21b^2)*A_0)-((7315c^4*7b)* A_{1_{corr}})-(7315c^4*A_{2_{corr}})-(26334c^5*7b)*A_0)-(26334c^5*A_{1_{corr}})-(74613c^6*A_0)$ |
| $A_{7_{corr}}$ | $A_7-(b^7*A_0)-(140a^3b*A_0)-(105a^2b*A_{2_{corr}})-(42ab*A_{4_{corr}})-(42ab^5*A_0)-(105ab^4*A_{1_{corr}})-(140ab^3*A_{2_{corr}})-(105ab^2*A_{3_{corr}})-(210a^2b^3*A_0)-(210a^2b^2*A_{1_{corr}})-(35a^3*A_{1_{corr}})-(21a^2*A_{3_{corr}})-(7a*A_{5_{corr}})-(7b^6*A_{1_{corr}})-(21b^5*A_{2_{corr}})-(35b^4*A_{3_{corr}})-(35b^3*A_{4_{corr}})-(21b^2*A_{5_{corr}})-(7b*A_{6_{corr}})-((22c*105a^2b)* A_{1_{corr}})-((22c*42ab)* A_{3_{corr}})-((22c*105ab^4)*A_0)-((22c*140ab^3)* A_{1_{corr}})-((22c*105ab^2)* A_{2_{corr}})-((22c*210a^2b^2)*A_0)-((22c*35a^3)*A_0)-((22c*21a^2)* A_{2_{corr}})-((22c*7a)* A_{4_{corr}})-((22c*7b^6)*A_0)-((22c*21b^5)* A_{1_{corr}})-((22c*35b^4)* A_{2_{corr}})-((22c*35b^3)* A_{3_{corr}})-((22c*21b^2)* A_{4_{corr}})-((22c*7b)* A_{5_{corr}})-(22c*A_{6_{corr}})-((231c^2*105a^2b)*A_0)-((231c^2*42ab)* A_{2_{corr}})-((231c^2*140ab^3)*A_0)-((231c^2*105ab^2)* A_{1_{corr}})-((231c^2*21a^2)* A_{1_{corr}})-((231c^2*7a)* A_{3_{corr}})-((231c^2*21b^5)*A_0)-((231c^2*35b^4)* A_{1_{corr}})-((231c^2*35b^3)* A_{2_{corr}})-((231c^2*21b^2)* A_{3_{corr}})-((231c^2*7b)* A_{4_{corr}})-(231c^2*A_{5_{corr}})-((1540c^3*42ab)* A_{1_{corr}})-((1540c^3*105ab^2)*A_0)-((1540c^3*21a^2)*A_0)-((1540c^3*7a)* A_{2_{corr}})-((1540c^3*35b^4)*A_0)-((1540c^3*35b3)* A_{1_{corr}})-((1540c^3*21b2)* A_{2_{corr}})-((1540c^3*7b)* A_{3_{corr}})-(1540c^3*A_{4_{corr}})-((7315c^4*42ab)*A_0)-((7315c^4*7a)* A_{1_{corr}})-((7315c^4*35b3)*A_0)-(7315c^4*21b^2)* A_{1_{corr}})-((7315c^4*7b)* A_{2_{corr}})-(7315c^4*A_{3_{corr}})-((26334c^5*7a)*A_0)-((26334c^5*21b^2)*A_0)-(26334c^5*7b)* A_{1_{corr}})-(26334c^5*A_{2_{corr}})-((74613c^6*7b)*A_0)- (74613c^6*A_{1_{corr}})- (170544c^7*A_0)$ |

**Table S2** Isotopologue fractions of metabolites implemented into metabolic network model, including the Monte Carlo simulated

| | **Biological Replicate 1** | | | | **Biological Replicate 2** | | | | **Biological Replicate 3** | | | |
|---|---|---|---|---|---|---|---|---|---|---|---|---|
| | Input areas (*before correction*) | **IF corrected %** | **IF corrected U** | **IF corrected rel.U** | Input areas *(before correction)* | **IF corrected %** | **IF corrected U** | **IF corrected rel.U** | Input areas *(before correction)* | **IF corrected %** | **IF corrected U** | **IF corrected rel.U** |
| PEP M | 7187 | **22.328** | **0.312** | **1.40%** | 4070 | **17.116** | **0.282** | **1.65%** | 4337 | **19.607** | **0.321** | **1.64%** |
| PEP M+1 | 26796 | **77.672** | **0.312** | **0.40%** | 20360 | **81.348** | **0.406** | **0.50%** | 18865 | **80.393** | **0.321** | **0.40%** |
| PEP M+2 | 191 | *-21.648* | *0.309* | *-1.43%* | 5262 | *-0.367* | *0.498* | *-135.46%* | 220 | *-21.579* | *0.321* | *-1.49%* |
| PEP M+3 | 124 | *-9.990* | *0.092* | *-0.92%* | 2917 | **1.535** | **0.269** | **17.51%** | 184 | *-9.833* | *0.107* | *-1.09%* |
| GAP M | 16889 | **22.179** | **0.254** | **1.1%** | 10200 | **17.5** | **0.23** | **1.3%** | 8485 | **17.832** | **0.240** | **1.3%** |
| GAP M+1 | 59006 | **71.467** | **0.495** | **0.7%** | 47030 | **75.8** | **0.52** | **0.7%** | 37982 | **74.983** | **0.534** | **0.7%** |
| GAP M+2 | 20733 | **4.884** | **0.456** | **9.3%** | 16298 | **5.0** | **0.49** | **9.7%** | 13614 | **5.892** | **0.496** | **8.4%** |
| GAP M+3 | 9773 | **1.469** | **0.183** | **12.4%** | 7917 | **1.7** | **0.20** | **11.9%** | 6325 | **1.293** | **0.213** | **16.5%** |
| 2PG M | 4838 | **33.4** | **0.5085** | **1.5%** | 1914 | **11.4** | **0.2794** | **2.5%** | 2181 | **18.9** | **0.4297** | **2.3%** |
| 2PG M+1 | 11267 | **66.6** | **0.5085** | **0.8%** | 15559 | **88.6** | **0.2794** | **0.3%** | 10029 | **80.6** | **0.8006** | **1.0%** |
| 2PG M+2 | 3270 | *-5.8* | *0.5839* | *-10.1%* | 2545 | *-16.5* | *0.5298* | *-3.2%* | 3560 | **0.5** | **0.7539** | **141.6%** |
| 2PG M+3 | 1886 | *-0.6* | *0.3720* | *-58.4%* | 1583 | *-7.4* | *0.3088* | *-4.2%* | 1815 | *-0.1* | *0.4375* | *-775.0%* |
| DHAP M | 58885 | **26.4** | **0.26** | **1.0%** | 36988 | **22.1** | **0.2** | **1.0%** | 30549 | **20.5** | **0.22** | **1.1%** |
| DHAP M+1 | 171641 | **69.7** | **0.46** | **0.7%** | 133360 | **73.5** | **0.5** | **0.6%** | 118357 | **73.9** | **0.48** | **0.6%** |
| DHAP M+2 | 58301 | **3.7** | **0.42** | **11.4%** | 45115 | **4.0** | **0.4** | **11.0%** | 41604 | **5.2** | **0.45** | **8.7%** |
| DHAP M+3 | 24830 | **0.2** | **0.14** | **64.1%** | 19758 | **0.4** | **0.1** | **38.7%** | 18189 | **0.5** | **0.15** | **31.5%** |
| 3PG M | 57203 | **20.4** | **0.218** | **1.1%** | 31769 | **7.6** | **0.091** | **1.2%** | 35850 | **15.3** | **0.174** | **1.1%** |
| 3PG M+1 | 242520 | **79.6** | **0.218** | **0.3%** | 397295 | **92.4** | **0.091** | **0.1%** | 210681 | **84.7** | **0.174** | **0.2%** |
| 3PG M+2 | 82359 | *-0.9* | *0.521* | *-57.1%* | 64116 | *-16.8* | *0.496* | *-2.9%* | 70005 | *-1.2* | *0.549* | *-46.6%* |
| 3PG M+3 | 36496 | *-2.5* | *0.213* | *-8.6%* | 29064 | *-10.3* | *0.178* | *-1.7%* | 32245 | *-2.5* | *0.225* | *-9.2%* |

| | Biological Replicate 1 | | | | Biological Replicate 2 | | | | Biological Replicate 3 | | | |
|---|---|---|---|---|---|---|---|---|---|---|---|---|
| | Input areas (*before correction*) | **IF corrected %** | **IF corrected U** | **IF corrected rel.U** | Input areas *(before correction)* | **IF corrected %** | **IF corrected U** | **IF corrected rel.U** | Input areas *(before correction)* | **IF corrected %** | **IF corrected U** | **IF corrected rel.U** |
| E4P M | 0 | **0.0** | **0.0000** | **-** | 0 | **0.0** | **0.0000** | **-** | 0 | **0.0** | **0.0000** | **-** |
| E4P M+1 | 1455 | **100.0** | **0.0000** | **0.0%** | 898 | **71.5** | **2.0080** | **2.8%** | 925 | **100.0** | **0.0000** | **0.0%** |
| E4P M+2 | 459 | *-2.9* | *1.8060* | *-63.3%* | 667 | **28.5** | **2.0080** | **7.0%** | 276 | *-4.5* | *2.1500* | *-47.7%* |
| E4P M+3 | 0 | *-18.7* | *0.1787* | *-1.0%* | 0 | *-23.2* | *0.3901* | *-1.7%* | 0 | *-18.7* | *0.1788* | *-1.0%* |
| E4P M+4 | 0 | *-4.4* | *0.0979* | *-2.2%* | 0 | *-8.5* | *0.2958* | *-3.5%* | 0 | *-4.4* | *0.0979* | *-2.2%* |
| R5P M | 8298 | **13.09** | **0.1883** | **1.4%** | 6924 | **9.99** | **0.1561** | **1.6%** | 5106 | **10.73** | **0.1778** | **1.7%** |
| R5P M+1 | 57540 | **85.18** | **0.2624** | **0.3%** | 63924 | **87.97** | **0.7092** | **0.8%** | 38603 | **76.54** | **0.7125** | **0.9%** |
| R5P M+2 | 25087 | *-0.09* | *0.7657* | *-828.7%* | 27864 | **0.12** | **0.7806** | **625.2%** | 21403 | **9.59** | **0.7218** | **7.5%** |
| R5P M+3 | 13721 | *-0.73* | *0.3930* | *-53.9%* | 15627 | *-0.35* | *0.3048* | *-86.1%* | 12976 | **3.14** | **0.3742** | **11.9%** |
| R5P M+4 | 5211 | **1.69** | **0.1953** | **11.5%** | 5738 | **1.59** | **0.1604** | **10.1%** | 4371 | *-0.41* | *0.2077* | *-50.6%* |
| R5P M+5 | 1756 | **0.03** | **0.0881** | **269.1%** | 2131 | **0.32** | **0.0895** | **27.6%** | 1291 | *-0.58* | *0.1141* | *-19.5%* |
| Rl5P M | 6800 | **13.60** | **0.2081** | **1.5%** | 4432 | **8.95** | **0.1545** | **1.7%** | 2647 | **8.11** | **0.1659** | **2.0%** |
| Rl5P M+1 | 44736 | **83.66** | **0.6909** | **0.8%** | 46460 | **90.02** | **0.2426** | **0.3%** | 26403 | **77.48** | **0.7535** | **1.0%** |
| Rl5P M+2 | 19641 | **0.13** | **0.7665** | **573.3%** | 19896 | *-0.51* | *0.8104* | *-159.8%* | 14918 | **10.61** | **0.7518** | **7.1%** |
| Rl5P M+3 | 11495 | **0.90** | **0.3307** | **36.6%** | 10228 | *-2.63* | *0.4064* | *-15.5%* | 9263 | **3.79** | **0.4192** | **11.1%** |
| Rl5P M+4 | 4099 | **1.35** | **0.1867** | **13.8%** | 3869 | **1.03** | **0.2067** | **20.1%** | 3012 | *-0.90* | *0.2375* | *-26.3%* |
| Rl5P M+5 | 1577 | **0.36** | **0.1066** | **29.8%** | 1263 | **0.01** | **0.0948** | **1488.1%** | 748 | *-1.25* | *0.1264* | *-10.1%* |

| | **Biological Replicate 1** | | | | **Biological Replicate 2** | | | | **Biological Replicate 3** | | | |
|---|---|---|---|---|---|---|---|---|---|---|---|---|
| | Input areas (*before correction*) | **IF corrected %** | **IF corrected U** | **IF corrected rel.U** | Input areas *(before correction)* | **IF corrected %** | **IF corrected U** | **IF corrected rel.U** | Input areas *(before correction)* | **IF corrected %** | **IF corrected U** | **IF corrected rel.U** |
| F6P M | 12292 | **9.8** | **0.1331** | **1.4%** | 10408 | **7.5** | **0.1105** | **1.5%** | 8059 | **6.8** | **0.1052** | **1.5%** |
| F6P M+1 | 49717 | **34.6** | **0.3787** | **1.1%** | 51064 | **33.0** | **0.3791** | **1.1%** | 43492 | **33.3** | **0.3789** | **1.1%** |
| F6P M+2 | 93411 | **53.6** | **0.4475** | **0.8%** | 106413 | **57.5** | **0.5808** | **1.0%** | 89332 | **56.4** | **0.5837** | **1.0%** |
| F6P M+3 | 47825 | *-1.5* | *0.6353* | *-42.5%* | 58596 | **1.5** | **0.6699** | **46.0%** | 50208 | **2.2** | **0.6634** | **30.0%** |
| F6P M+4 | 25697 | *-1.0* | *0.3584* | *-35.5%* | 31496 | *-0.5* | *0.2966* | *-59.8%* | 28629 | **1.0** | **0.3139** | **32.3%** |
| F6P M+5 | 11705 | **2.0** | **0.1829** | **9.2%** | 12003 | **0.5** | **0.1641** | **32.5%** | 10746 | **0.3** | **0.1742** | **55.0%** |
| F6P M+6 | 3799 | *-0.4* | *0.0769* | *-19.1%* | 3521 | *-0.4* | *0.0718* | *-17.0%* | 3398 | *-0.3* | *0.0839* | *-24.2%* |
| G6P M | 135074 | **7.5** | **0.0925** | **1.2%** | 61876 | **4.6** | **0.0589** | **1.3%** | 56931 | **4.7** | **0.0579** | **1.2%** |
| G6P M+1 | 423549 | **19.7** | **0.2268** | **1.2%** | 281296 | **18.6** | **0.2176** | **1.2%** | 250380 | **18.2** | **0.2029** | **1.1%** |
| G6P M+2 | 1523656 | **72.1** | **0.6795** | **0.9%** | 1170308 | **76.2** | **0.7093** | **0.9%** | 1046916 | **75.2** | **0.7001** | **0.9%** |
| G6P M+3 | 805286 | **0.7** | **0.7525** | **103.8%** | 616549 | **0.5** | **0.7873** | **147.0%** | 550890 | **0.5** | **0.7785** | **158.4%** |
| G6P M+4 | 441285 | *-1.6* | *0.2773* | *-17.4%* | 340706 | *-1.7* | *0.2868* | *-16.9%* | 343251 | **1.5** | **0.3063** | **20.4%** |
| G6P M+5 | 152261 | *-0.6* | *0.1436* | *-25.3%* | 116695 | *-0.6* | *0.1476* | *-22.8%* | 108089 | *-1.1* | *0.1566* | *-14.4%* |
| G6P M+6 | 46143 | *-0.5* | *0.0483* | *-9.7%* | 36908 | *-0.4* | *0.0507* | *-11.9%* | 34085 | *-0.8* | *0.0871* | *-10.7%* |
| S7P M | 11269 | **10.40** | **0.1578** | **1.5%** | 7028 | **7.41** | **0.1228** | **1.7%** | 5353 | **7.56** | **0.1336** | **1.8%** |
| S7P M+1 | 78733 | **66.48** | **0.7571** | **1.1%** | 69047 | **68.38** | **0.7817** | **1.1%** | 47172 | **62.17** | **0.7401** | **1.2%** |
| S7P M+2 | 69363 | **20.38** | **0.7962** | **3.9%** | 62804 | **22.64** | **0.8078** | **3.6%** | 47476 | **27.16** | **0.7703** | **2.8%** |
| S7P M+3 | 46702 | **2.74** | **0.5387** | **19.6%** | 41163 | **1.40** | **0.5684** | **40.6%** | 32009 | **3.03** | **0.6073** | **20.0%** |
| S7P M+4 | 21976 | *-0.53* | *0.3217* | *-60.4%* | 19816 | *-0.15* | *0.3330* | *-219.0%* | 14852 | *-1.86* | *0.3538* | *-19.0%* |
| S7P M+5 | 8984 | *-0.14* | *0.1987* | *-141.9%* | 7643 | *-0.25* | *0.2029* | *-80.5%* | 6108 | *-0.67* | *0.2209* | *-33.1%* |
| S7P M+6 | 3007 | *-0.06* | *0.0846* | *-153.4%* | 2797 | **0.17** | **0.0886** | **50.7%** | 2182 | *-0.11* | *0.1021* | *-93.7%* |
| S7P M+7 | 819 | *-0.07* | *0.0379* | *-52.4%* | 835 | *-0.02* | *0.0480* | *-293.1%* | 723 | **0.08** | **0.0486** | **58.4%** |

**Table S3** Isotopologues from metabolites in the pentose phosphate pathway suggesting missing reactions in the model or measurement bias. The metabolites are expected to be part of a linear pathway and show the same labeling patterns, but differences of more than 10% are observed

| | | Average | SD |
|---|---|---|---|
| Glucose-6-Phosphate | G6P_M | 3.0 | 1.2 |
| | G6P_M1 | 17.6 | 0.7 |
| | G6P_M2 | 77.8 | 0.9 |
| | G6P_M3 | 1.6 | 2.8 |
| | G6P_M4 | 0.0 | 0.0 |
| | G6P_M5 | 0.0 | 0.0 |
| | G6P_M6 | 0.0 | 0.0 |
| 6-phosphogluconate | 6PGA_M | 14.8 | 2.5 |
| | 6PGA_M1 | 20.9 | 2.8 |
| | 6PGA_M2 | 63.8 | 1.6 |
| | 6PGA_M3 | 0.2 | 0.4 |
| | 6PGA_M4 | 0.0 | 0.1 |
| | 6PGA_M5 | 0.1 | 0.2 |
| | 6PGA_M6 | 0.0 | 0.0 |

**Table S4** Measured and simulated isotopologue fractions handling uncertainties using different strategies

| | Measurement | Standard deviation (SD) | Simulated values using all measurements | Simulated values using measurements >2% | Simulated values using measurements > 2% and 3 fold SD increase to cover biological variance | Simulated values using measurements > 2% and 3-fold SD increase to cover biological variance except for F6P and G6P (M+2 andM+3) |
|---|---|---|---|---|---|---|
| GAP_M | 0.192 | 0.021 | 0.157 | 0.157 | 0.157 | 0.154 |
| GAP_M1 | 0.741 | 0.019 | 0.825 | 0.825 | 0.825 | 0.828 |
| GAP_M2 | 0.053 | 0.004 | 0.018 | 0.018 | 0.018 | 0.018 |
| GAP_M3 | 0.015 | 0.002 | 0.000 | | | |
| DHAP_M | 0.230 | 0.025 | 0.246 | 0.246 | 0.246 | 0.246 |
| DHAP_M1 | 0.724 | 0.019 | 0.738 | 0.738 | 0.738 | 0.738 |
| DHAP_M2 | 0.043 | 0.006 | 0.016 | 0.016 | 0.016 | 0.016 |
| DHAP_M3 | 0.004 | 0.001 | 0.000 | | | |
| E4P_M | 0.000 | 0.152 | 0.041 | | | |
| E4P_M1 | 0.905 | 0.134 | 0.929 | 0.929 | 0.929 | 0.932 |
| E4P_M2 | 0.071 | 0.152 | 0.030 | 0.030 | 0.030 | 0.030 |
| E4P_M3 | 0.000 | 0.021 | 0.000 | | | |
| E4P_M4 | 0.000 | 0.019 | 0.000 | | | |
| RI5P_M | 0.102 | 0.024 | 0.038 | 0.038 | 0.038 | 0.037 |
| RI5P_M1 | 0.837 | 0.051 | 0.922 | 0.922 | 0.922 | 0.922 |
| RI5P_M2 | 0.034 | 0.051 | 0.040 | 0.040 | 0.040 | 0.040 |
| RI5P_M3 | 0.007 | 0.026 | 0.001 | | | |
| RI5P_M4 | 0.005 | 0.010 | 0.000 | | | |
| RI5P_M5 | 0.000 | 0.007 | 0.000 | | | |
| R5P_M | 0.113 | 0.013 | 0.039 | 0.039 | 0.039 | 0.037 |
| R5P_M1 | 0.832 | 0.049 | 0.920 | 0.921 | 0.921 | 0.922 |
| R5P_M2 | 0.032 | 0.045 | 0.040 | 0.040 | 0.040 | 0.040 |
| R5P_M3 | 0.007 | 0.017 | 0.001 | | | |
| R5P_M4 | 0.010 | 0.010 | 0.000 | | | |
| R5P_M5 | 0.000 | 0.004 | 0.000 | | | |
| F6P_M | 0.080 | 0.013 | 0.047 | | | |
| F6P_M1 | 0.336 | 0.007 | 0.352 | 0.047 | 0.047 | 0.046 |
| F6P_M2 | 0.558 | 0.016 | 0.575 | 0.352 | 0.352 | 0.344 |
| F6P_M3 | 0.007 | 0.016 | 0.024 | 0.576 | 0.576 | 0.585 |
| F6P_M4 | 0.000 | 0.008 | 0.000 | | | |
| F6P_M5 | 0.009 | 0.007 | 0.000 | | | |
| F6P_M6 | 0.000 | 0.000 | 0.000 | | | |
| S7P_M | 0.085 | 0.014 | 0.028 | 0.028 | 0.028 | 0.027 |
| S7P_M1 | 0.657 | 0.026 | 0.669 | 0.669 | 0.668 | 0.669 |
| S7P_M2 | 0.234 | 0.028 | 0.288 | 0.289 | 0.289 | 0.290 |
| S7P_M3 | 0.024 | 0.007 | 0.014 | 0.014 | 0.014 | 0.015 |
| S7P_M4 | 0.000 | 0.007 | 0.000 | | | |
| S7P_M5 | 0.000 | 0.002 | 0.000 | | | |
| S7P_M6 | 0.000 | 0.001 | 0.000 | | | |

| | | | | | | |
|---|---|---|---|---|---|---|
| S7P_M7 | 0.000 | 0.001 | 0.000 | | | |
| G6P_M | 0.030 | 0.012 | 0.022 | 0.022 | 0.022 | 0.022 |
| G6P_M1 | 0.176 | 0.007 | 0.175 | 0.175 | 0.175 | 0.172 |
| G6P_M2 | 0.778 | 0.009 | 0.769 | 0.769 | 0.769 | 0.772 |
| G6P_M3 | 0.016 | 0.028 | 0.033 | | | |
| G6P_M4 | 0.000 | 0.028 | 0.001 | | | |
| G6P_M5 | 0.000 | 0.028 | 0.000 | | | |
| G6P_M6 | 0.000 | 0.028 | 0.000 | | | |
